# Consensus clustering for Bayesian mixture models

**DOI:** 10.1186/s12859-022-04830-8

**Published:** 2022-07-21

**Authors:** Stephen Coleman, Paul D. W. Kirk, Chris Wallace

**Affiliations:** 1 MRC Biostatistics Unit, University of Cambridge, Cambridge, UK; 2 Cambridge Institute of Therapeutic Immunology and Infectious Disease, University of Cambridge, Cambridge, UK

**Keywords:** Cluster analysis, Cell cycle, Ensemble learning, Integrative clustering

## Abstract

**Background:**

Cluster analysis is an integral part of precision medicine and systems biology, used to define groups of patients or biomolecules. Consensus clustering is an ensemble approach that is widely used in these areas, which combines the output from multiple runs of a non-deterministic clustering algorithm. Here we consider the application of consensus clustering to a broad class of heuristic clustering algorithms that can be derived from Bayesian mixture models (and extensions thereof) by adopting an early stopping criterion when performing sampling-based inference for these models. While the resulting approach is non-Bayesian, it inherits the usual benefits of consensus clustering, particularly in terms of computational scalability and providing assessments of clustering stability/robustness.

**Results:**

In simulation studies, we show that our approach can successfully uncover the target clustering structure, while also exploring different plausible clusterings of the data. We show that, when a parallel computation environment is available, our approach offers significant reductions in runtime compared to performing sampling-based Bayesian inference for the underlying model, while retaining many of the practical benefits of the Bayesian approach, such as exploring different numbers of clusters. We propose a heuristic to decide upon ensemble size and the early stopping criterion, and then apply consensus clustering to a clustering algorithm derived from a Bayesian integrative clustering method. We use the resulting approach to perform an integrative analysis of three ’omics datasets for budding yeast and find clusters of co-expressed genes with shared regulatory proteins. We validate these clusters using data external to the analysis.

**Conclustions:**

Our approach can be used as a wrapper for essentially any existing sampling-based Bayesian clustering implementation, and enables meaningful clustering analyses to be performed using such implementations, even when computational Bayesian inference is not feasible, e.g. due to poor exploration of the target density (often as a result of increasing numbers of features) or a limited computational budget that does not along sufficient samples to drawn from a single chain. This enables researchers to straightforwardly extend the applicability of existing software to much larger datasets, including implementations of sophisticated models such as those that jointly model multiple datasets.

## Background

From defining a taxonomy of disease to creating molecular sets, grouping items can help us to understand and make decisions using complex biological data. For example, grouping patients based upon disease characteristics and personal omics data may allow the identification of more homogeneous subgroups, enabling stratified medicine approaches. Defining and studying molecular sets can improve our understanding of biological systems as these sets are more interpretable than their constituent members [1], and study of their interactions and perturbations may have ramifications for diagnosis and drug targets [2, 3]. The act of identifying such groups is referred to as *cluster analysis*. Many traditional methods such as *K*-means clustering [4, 5] condition upon a fixed choice of *K*, the number of clusters. These methods are often heuristic in nature, relying on rules of thumb to decide upon a final value for *K*. For example, different choices of *K* are compared under some metric such as silhouette [6] or the within-cluster sum of squared errors (**SSE**) as a function of *K*. Moreover, *K*-means clustering can exhibit sensitivity to initialisation, necessitating multiple runs in practice [7].

Another common problem is that traditional methods offer no measure of the stability or robustness of the final clustering. Returning to the stratified medicine example of clustering patients, there might be individuals that do not clearly belong to any one particular cluster; however if only a point estimate is obtained, this information is not available. Ensemble methods address this problem, as well as reducing sensitivity to initialisation. These approaches have had great success in supervised learning, most famously in the form of Random Forest [8] and boosting [9]. In clustering, consensus clustering [10] is a popular method which has been implemented in R [11] and to a variety of methods [12, 13] and been applied to problems such as cancer subtyping [14, 15] and identifying subclones in single cell analysis [16]. Consensus clustering uses *W* runs of some base clustering algorithm (such as *K*-means). These *W* proposed partitions are commonly compiled into a *consensus matrix*, the (*i*, *j*)th entries of which contain the proportion of model runs for which the *i*th and *j*th individuals co-cluster (for this and other definitions see section 1 of the Additional file 1), although this step is not fundamental to consensus clustering and there is a large body of literature aimed at interpreting a collection of partitions (see, e.g., [17–19]). This consensus matrix provides an assessment of the stability of the clustering. Furthermore, ensembles can offer reductions in computational runtime because the individual members of the ensemble are often computationally inexpensive to fit (e.g, because they are fitted using only a subset of the available data) and because the learners in most ensemble methods are independent of each other and thus enable use of a parallel environment for each of the quicker model runs [20].

Traditional clustering methods usually condition upon a fixed choice of *K*, the number of clusters with the choice of *K* being a difficult problem in itself. In consensus clustering, Monti et al. [10] proposed methods for choosing *K* using the consensus matrix and Ünlü et al. [21] offer an approach to estimating *K* given the collection of partitions, but each clustering run uses the same, fixed, number of clusters. An alternative clustering approach, mixture modelling, embeds the cluster analysis within a formal, statistical framework [22]. This means that models can be compared formally, and problems such as the choice of *K* can be addressed as a model selection problem [23]. Moreover, *Bayesian mixture models* can be used to try to directly infer *K* from the data. Such inference can be performed through use of a Dirichlet Process mixture model [24–26], a mixture of finite mixture models [27, 28] or an over-fitted mixture model [29]. The Bayesian model also assesses the uncertainty in the cluster allocations, and if *K* is treated as a random variable uncertainty about the value of *K* propagates through to the clustering. Furthermore, the Bayesian hierarchical modelling framework enables extrapolating the mixture model to capture more complex dependencies, for example, integrative clustering methods tailored for multi-omics analysis [30–32]. Bayesian clustering methods have a history of successful application to a diverse range of biological problems such as finding clusters of gene expression profiles [33], cell types in flow cytometry [34, 35] or scRNAseq experiments [36], and estimating protein localisation [37].

Markov chain Monte Carlo (MCMC) methods are the most common tool for performing computational Bayesian inference. They guarantee an exact description of the posterior distribution in the limit of infinite iterations in contrast to Variational Inference [38]. In Bayesian clustering, they are used to draw a collection of clustering partitions from the posterior distribution. In practice MCMC methods may explore the parameter space very inefficiently despite their ergodic properties. As the number of features/measurements increases this inefficiency can become pathological with chains prone to becoming stuck in local posterior modes preventing convergence in any feasible period of runtime (see, e.g., the Supplementary Materials of [39]; this problem is frequently referred to as poor mixing within the chain. There is a rich zoo of MCMC methods designed to overcome the different limitations of the most basic samplers. For example, there are MCMC methods that use parallel chains to improve the scalability with an increasing number of observations, such as Consensus Monte Carlo [40–42]. Consensus Monte Carlo methods subsample the original dataset and run separate chains on each smaller dataset. In this way they can use a far smaller quantity of data for each Monte Carlo algorithm and treat each chain as embarrassingly parallel enabling simultaneous model runs across machines, with the samples then averaged across chains. This parallelisation and reduced dataset size offers a significant reduction in runtime for large *N* datasets. Another method designed to improve scaling to large datasets, is stochastic gradient MCMC (SGMCMC [43]). This uses a subset of the data in each sampling iteration and has provable guarantees [44]. However, SGMCMC converges at a slower rate than traditional MCMC algorithms and remains computationally costly [45, 46]. While these methods help in scaling to large N data, they are less helpful in situations where we have high-dimension but only moderate sample size, such as frequently arises in analysis of ’omics data. Other methods such as coupling [47] use multiple chains to reduce the bias of the Monte Carlo estimate.

Other MCMC methods make efforts to overcome the problem of poor mixing at the cost of increased computational cost per iteration [48]. In clustering models introducing split-merge moves into the sampler are the most common examples of such bold exploration moves (see, e.g., [49–52]). However, these methods are difficult to implement and frequently propose many rejected moves, thereby increasing computational cost without necessarily guaranteeing full exploration of the target density in any finite amount of time. Furthermore, most available Bayesian clustering methods are implemented using a basic Gibbs sampler and would require reimplementation to exploit more scalable samplers, a costly investment of time and effort. Ideally these existing implementations could be used despite their simple sampler.

There also exists a range of alternative clustering methods that are designed or have been extended to scale well with increasing sample size, e.g., *k*-means clustering [53, 54], spectral clustering[55, 56], density-based clustering[57, 58], etc., any of which could be used within a consensus clustering wrapper. However, while these methods have better scalability than sampling based clustering methods, they suffer from a lack of flexibility. They do not, in general, have the ability to explore multiple values of *K* in a single model run, it is not easy to extend these methods to the multiple dataset problem and they are often restricted to a specific data type.

As described above, sampling-based Bayesian clustering methods are flexible, capable of modelling complex dependencies and the number of clusters present. However, they suffer from prohibitive runtimes and poor exploration in high-dimensional data (i.e., large *P* data). This limits the consistency of their inference in biomedical applications where data is often very high dimensional with only moderate sample size (for some discussion around stability in clustering, please see [59–61]). Motivated by this, we aim to develop a general and straightforward procedure that exploits the flexibility of Bayesian model based clustering methods but improves their performance under a constrained computational budget without requiring reimplementation. Specifically, we make use of existing sampling-based Bayesian clustering implementations, but only run them for a fixed (and relatively small) number of iterations, stopping before they have converged to their target stationary distribution. We initialise each chain with a random draw from an uninformative prior distribution on the space of partitions and then collect the final sampled partition. Doing this repeatedly, we obtain an ensemble of clustering partitions which has large variety in its initialisation. We use this set to perform consensus clustering, constructing a consensus matrix (thereby avoiding the label-switching problem) which describes uncertainty about the latent structure in the data. This can be used to infer a point estimate clustering. We propose a heuristic for deciding upon the ensemble size (the number of learners used, *W*) and the ensemble depth (the number of iterations, *D*), inspired by the use of scree plots in Principal Component Analysis (**PCA**; [62]). We hope to show a way of scaling Bayesian mixture models and their extensions with increasing numbers of features that can explore a range of *K* in a single model run and can be tailored to specific properties of a given dataset. We note that, despite the similarity in names, our consensus clustering approach for Bayesian mixture models is very different to the consensus Monte Carlo approach of Ni et al. [41], which was designed to enable Bayesian mixture models to scale to large *N* datasets. Our approach leans into the ensemble framework of Monti et al. [10]; we consider the case that our individual chains are too short to have converged and in which case the inference is non-Bayesian, in contrast to Consensus Monte Carlo. Our primary aim is to mitigate the problem of poor mixing which tends to emerge when the data has a high numbers of features relative to the sample size and individual chains struggle to converge in a reasonable runtime, and to enable the use of complex models such as arise in multi-view or integrative analyses for which each iteration of the MCMC algorithm is slow even for small sample data and running a long chain might not be feasible.

We show via simulation that our approach can successfully identify meaningful clustering structures even when chains are very short. We then illustrate the use of our approach to extend the applicability of existing Bayesian clustering implementations, using as a case study the Multiple Dataset Integration (**MDI** [30] section 2 of the Additional file 1) model for Bayesian integrative clustering applied to real data. While the simulation results serve to validate our method, it is important to also evaluate methods on real data which may represent more challenging problems. For our real data, we use three ’omics datasets relating to the cell cycle of *Saccharomyces cerevisiae* with the aim of inferring clusters of genes across datasets. As there is no ground truth available, we then validate these clusters using knowledge external to the analysis.

## Material and methods

### Consensus clustering for Bayesian mixture models

We apply consensus clustering to MCMC based Bayesian clustering models using the method described in algorithm 1.
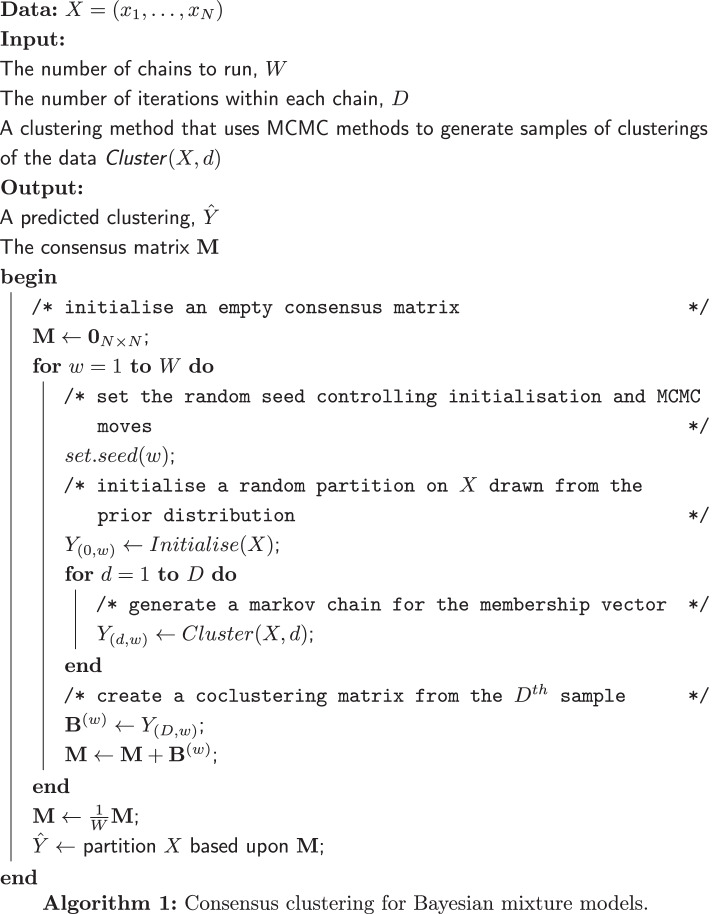


Our application of consensus clustering has two main parameters at the ensemble level, the chain depth, *D*, and ensemble width, *W*. We infer a point clustering from the consensus matrix using the maxpear function [63] from the R package mcclust [64] which maximises the posterior expected adjusted Rand index between the true clustering and point estimate if the matrix is composed of samples drawn from the posterior distribution (section 3 of the Additional file 1 for details). There are alternative choices of methods to infer a point estimate which minimise different loss functions (see, e.g., [65–67]).

#### Determining the ensemble depth and width

As our ensemble sidesteps the problem of convergence within each chain, we need an alternative stopping rule for growing the ensemble in chain depth, *D*, and number of chains, *W*. We propose a heuristic based upon the consensus matrix to decide if a given value of *D* and *W* are sufficient. We suspect that increasing *W* and *D* might continuously improve the performance of the ensemble (implicitly assuming that the Bayesian posterior distribution is the optimal description of the parameter of interest, an assumption that does not always hold, see, e.g. [68–70], but we observe in our simulations that these changes will become smaller and smaller for greater values, eventually converging for each of *W* and *D* (consider that if both $W \rightarrow \infty$ and $D \rightarrow \infty$ the ergodic properties of the MCMC sampler should emerge, exactly describing the entire posterior distribution). We notice that this behaviour is analogous to PCA in that where for consensus clustering some improvement might always be expected for increasing chain depth or ensemble width, more variance will be captured by increasing the number of components used in PCA. However, increasing this number beyond some threshold has diminishing returns, diagnosed in PCA by a scree plot. Following from this, we recommend, for some set of ensemble parameters, $D' = \{d_1, \ldots , d_I\}$ and $W'=\{w_1, \ldots , w_J\}$, find the mean absolute difference of the consensus matrix for the $d_i$th iteration from $w_j$ chains to that for the $d_{(i-1)}$th iteration from $w_j$ chains and plot these values as a function of chain depth, and the analogue for sequential consensus matrices for increasing ensemble width and constant depth.

If this heuristic is used, we believe that the consensus matrix and the resulting inference should be stable (see, e.g., [59, 60]), providing a robust estimate of the clustering. In contrast, if there is still strong variation in the consensus matrix for varying chain length or number, then we believe that the inferred clustering is influenced significantly by the random initialisation and that the inferred partition is unlikely to be stable for similar datasets or reproducible for a random choice of seeds.

### Simulation study

**Fig. 1:**
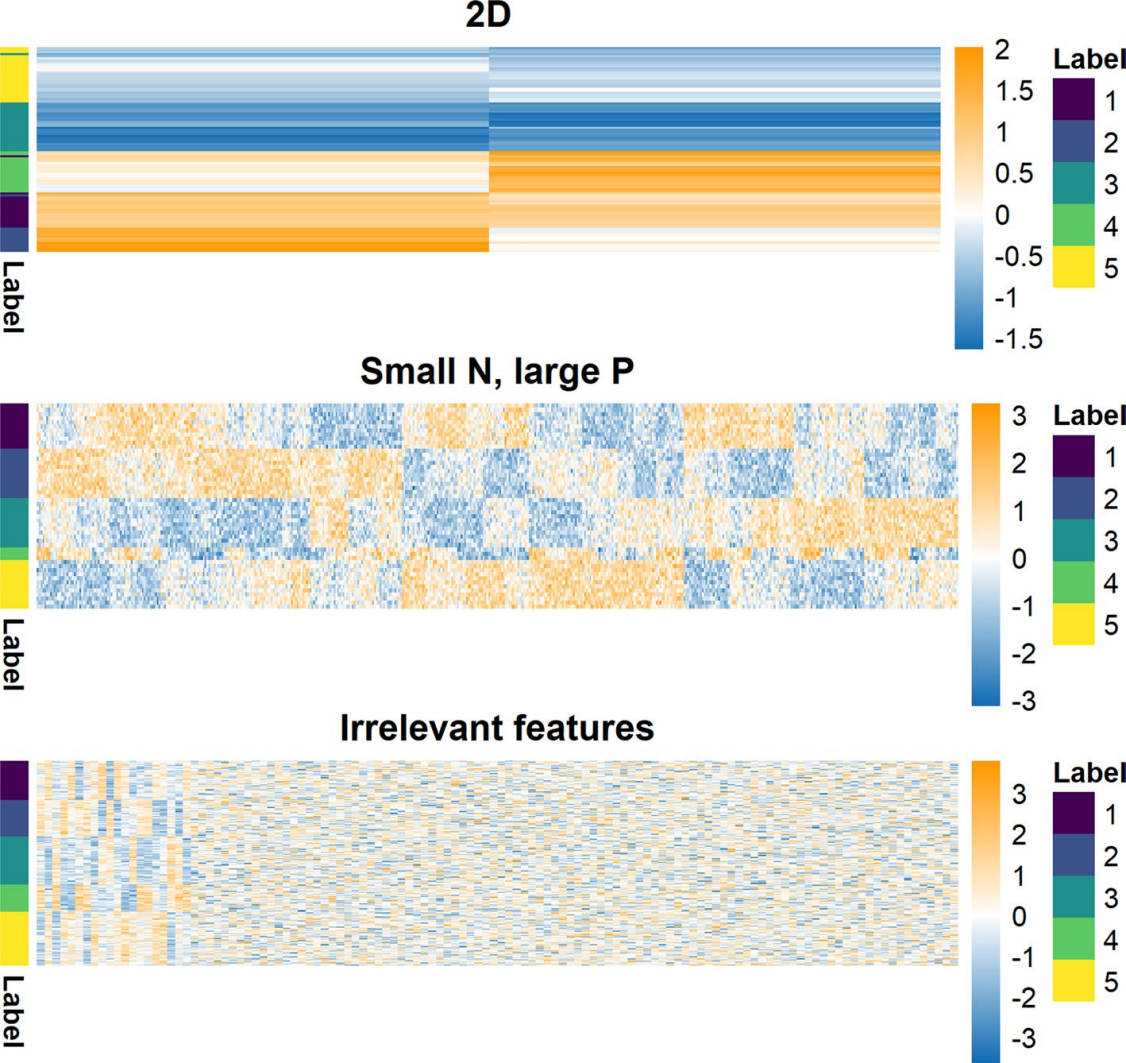
Example of generated datasets. Each row is an item being clustered and each column a feature of generated data. The 2D dataset (which is ordered by hierarchical clustering here) should enable proper mixing of chains in the MCMC. The small *N*, large *P* case has clear structure (observable by eye). This is intended to highlight the problems of poor mixing due to high dimensions even when the generating labels are quite identifiable. In the irrelevant features case, the structure is clear in the relevant features (on the left-hand side of this heatmap). This setting is intended to test how sensitive each approach is to noise.

We use a finite mixture with independent features as the data generating model within the simulation study. Within this model there exist “irrelevant features” [71] that have global parameters rather than cluster specific parameters. The generating model is

1 $$\begin{aligned} p(X, c, \theta , \pi | K) =&\nonumber \\ p(K) p(\pi |K)&p(\theta |K) \prod _{i=1}^N p (c_i | \pi , K) \prod _{p=1}^P p(x_{ip} | c_i, \theta _{c_ip})^ {\phi _p} p(x_{ip} | \theta _p) ^ {(1 - \phi _p)} \end{aligned}$$

for data $X=(x_1, \ldots , x_N)$, cluster label or allocation variable $c=(c_1, \ldots , c_N)$, cluster weight $\pi =(\pi _1, \ldots , \pi _K)$, *K* clusters and the relevance variable, $\phi \in \{0, 1\}$ with $\phi _p=1$ indicating that the *p*th feature is relevant to the clustering. We used a *Gaussian* density, so $\theta _{kp} = (\mu _{kp}, \sigma ^2_{kp})$. We defined three scenarios and simulated 100 datasets in each (Fig. 1 and Table 1). Additional details of the simulation process and additional scenarios are included in section 4.1 of the Additional file 1.

**Table 1:** Parameters defining the simulation scenarios as used in generating data and labels

Scenario	*N*	$P_s$	$P_n$	*K*	$\Delta \mu$	$\sigma ^2$	$\pi$
2D	100	2	0	5	3.0	1	$(\frac{1}{5}, \frac{1}{5}, \frac{1}{5}, \frac{1}{5}, \frac{1}{5})$
Small N, large P	50	500	0	5	1.0	1	$(\frac{1}{5}, \frac{1}{5}, \frac{1}{5}, \frac{1}{5}, \frac{1}{5})$
Irrelevant features	200	20	100	5	1.0	1	$(\frac{1}{5}, \frac{1}{5}, \frac{1}{5}, \frac{1}{5}, \frac{1}{5})$

$\Delta \mu$ is the distance between neighbouring cluster means within a single feature. The number of relevant features ($P_s$) is $\sum _p \phi _p$, and $P_n = P - P_s$

In each of these scenarios we apply a variety of methods (listed below) and compare the inferred point clusterings to the generating labels using the Adjusted Rand Index (**ARI**, [72]).

- Mclust, a maximum likelihood implementation of a finite mixture of Gaussian densities (for a range of modelled clusters, *K*),
- 10 chains of 1 million iterations, thinning to every thousandth sample for the overfitted Bayesian mixture of Gaussian densities, and
- A variety of consensus clustering ensembles defined by inputs of *W* chains and *D* iterations within each chain (see algorithm 1) with $W \in \{1, 10, 30, 50, 100\}$ and $D \in \{1, 10, 100, 1000, 10{,}000\}$ where the base learner is an overfitted Bayesian mixture of Gaussian densities.

Note that none of the applied methods include a model selection step and as such there is no modelling of the relevant variables. This and the unknown value of *K* is what separates the models used and the generating model described in Eq. (1). More specifically, the likelihood of a point $X_n$ for each method is

2 $$\begin{aligned} p(X_n | \mu , \Sigma , \pi )&= \sum _{k=1}^K \pi _k p(X_n | \mu _k, \Sigma _k), \end{aligned}$$

where $p(X_n | \mu _k, \Sigma _k)$ is the probability density function of the multivariate Gaussian distribution parameterised by a mean vector, $\mu _k$, and a covariance matrix, $\Sigma _k$, and $\pi _k$ is the component weight such that $\sum _{k=1}^K\pi _k = 1$. The implementation of the Bayesian mixture model restricts $\Sigma _k$ to be a diagonal matrix while Mclust models a number of different covariance structures. Note that while we use the overfitted Bayesian mixture model, this is purely from convenience and we expect that a true Dirichlet Process mixture or a mixture of mixture models would display similar behaviour in an ensemble.

The ARI is a measure of similarity between two partitions, $c_1, c_2$, corrected for chance, with 0 indicating $c_1$ is no more similar to $c_2$ than a random partition would be expected to be and a value of 1 showing that $c_1$ and $c_2$ perfectly align. Details of the methods in the simulation study can be found in sections 4.2, 4.3 and 4.4 of the Additional file 1.

#### Mclust

Mclust [73] is a function from the R package mclust. It estimates Gaussian mixture models for *K* clusters based upon the maximum likelihood estimator of the parameters. It initialises upon a hierarchical clustering of the data cut to *K* clusters. A range of choices of *K* and different covariance structures are compared and the “best” selected using the Bayesian information criterion [74] (details in section 4.2 of the Additional file 1).

#### Bayesian inference

To assess within-chain convergence of our Bayesian inference we use the Geweke *Z*-score statistic [75]. Of the chains that appear to behave properly we then asses across-chain convergence using ${\hat{R}}$ [76] and the recent extension provided by [77]. If a chain has reached its stationary distribution the Geweke *Z*-score statistic is expected to be normally distributed. Normality is tested for using a Shapiro–Wilks test [78]. If a chain fails this test (i.e., the associated *p* value is less than 0.05), we assume that it has not achieved stationarity and it is excluded from the remainder of the analysis. The samples from the remaining chains are then pooled and a posterior similarity matrix (**PSM**) constructed. We use the maxpear function to infer a point clustering. For more details see section 4.3 of the Additional file 1.

### Analysis of the cell cycle in budding yeast

#### Datasets

The cell cycle is crucial to biological growth, repair, reproduction, and development [79–81] and is highly conserved among eukaryotes [81]. This means that understanding of the cell cycle of *S. cerevisiae* can provide insight into a variety of cell cycle perturbations including those that occur in human cancer [80, 82] and ageing [83]. We aim to create clusters of genes that are co-expressed, have common regulatory proteins and share a biological function. To achieve this, we use three datasets that were generated using different ’omics technologies and target different aspects of the molecular biology underpinning the cell cycle process.

- Microarray profiles of RNA expression from [84], comprising measurements of cell-cycle-regulated gene expression at 5-min intervals for 200 min (up to three cell division cycles) and is referred to as the **time course** dataset. The cells are synchronised at the START checkpoint in late G1-phase using alpha factor arrest [84]. We include only the genes identified by [84] as having periodic expression profiles.
- Chromatin immunoprecipitation followed by microarray hybridization (**ChIP-chip**) data from [85]. This dataset discretizes *p* values from tests of association between 117 DNA-binding transcriptional regulators and a set of yeast genes. Based upon a significance threshold these *p* values are represented as either a 0 (no interaction) or a 1 (an interaction).
- Protein–protein interaction (**PPI**) data from BioGrid [86]. This database consists of of physical and genetic interactions between gene and gene products, with interactions either observed in high throughput experiments or computationally inferred. The dataset we used contained 603 proteins as columns. An entry of 1 in the (*i*, *j*)th cell indicates that the *i*th gene has a protein product that is believed to interact with the *j*th protein.

The datasets were reduced to the 551 genes with no missing data in the PPI and ChIP-chip data, as in [30].

#### Multiple dataset integration

We applied consensus clustering to MDI for our integrative analysis. Details of MDI are in section 2.2 of the Additional file 1, but in short MDI jointly models the clustering in each dataset, inferring individual clusterings for each dataset. These partitions are informed by similar structure in the other datasets, with MDI learning this similarity as it models the partitions. The model does not assume global structure. This means that the similarity between datasets is not strongly assumed in our model; individual clusters or genes that align across datasets are based solely upon the evidence present in the data and not due to strong modelling assumptions. Thus, datasets that share less common information can be included without fearing that this will warp the final clusterings in some way.

The datasets were modelled using a mixture of Gaussian processes in the time course dataset and Multinomial distributions in the ChIP-chip and PPI datasets.

## Results

### Simulated data

We use the ARI between the generating labels and the inferred clustering of each method to be our metric of predictive performance.

**Fig. 2:**
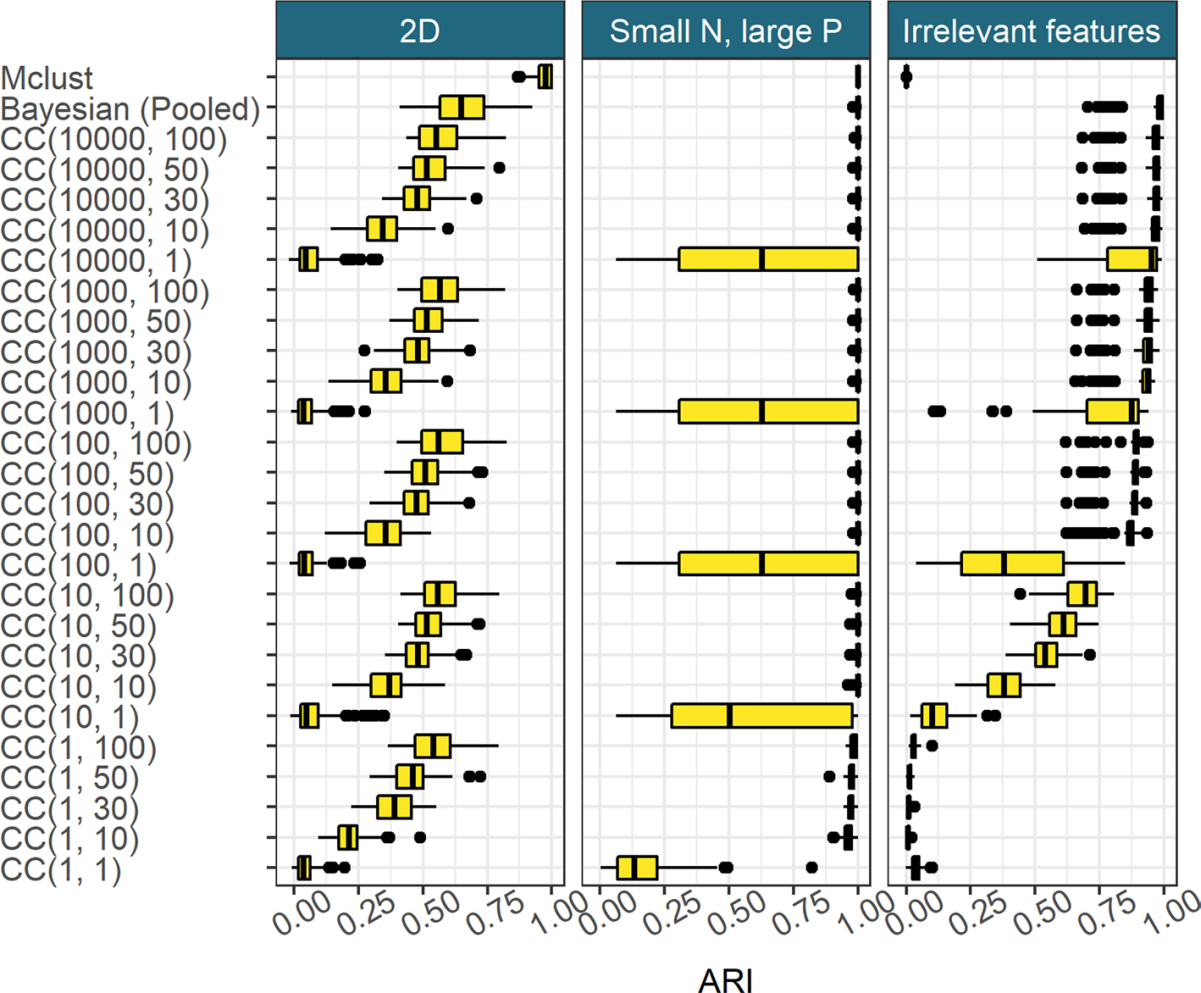
Model performance in the 100 simulated datasets for each scenario, defined as the ARI between the generating labels and the inferred clustering. *CC*(*d*, *w*) denotes consensus clustering using the clustering from the *d*th iteration from *w* different chains

In Fig. 2, we see Mclust performs very well in the 2D and Small *N*, large *P* scenarios, correctly identifying the true structure. However, the irrelevant features scenario sees a collapse in performance, Mclust is blinded by the irrelevant features and identifies a clustering of *K* = 1.

The pooled samples from multiple long chains performs very well across all scenarios and appears to act as an upper bound on the more practical implementations of consensus clustering.

Consensus clustering does uncover some of the generating structure in the data, even using a small number of short chains. With sufficiently large ensembles and chain depth, consensus clustering is close to the pooled Bayesian samples in predictive performance. It appears that for a constant chain depth increasing the ensemble width used follows a pattern of diminishing returns. There are strong initial gains for a greater ensemble width, but the improvement decreases for each successive chain. A similar pattern emerges in increasing chain length for a constant number of chains (Fig. 2).

**Fig. 3:**
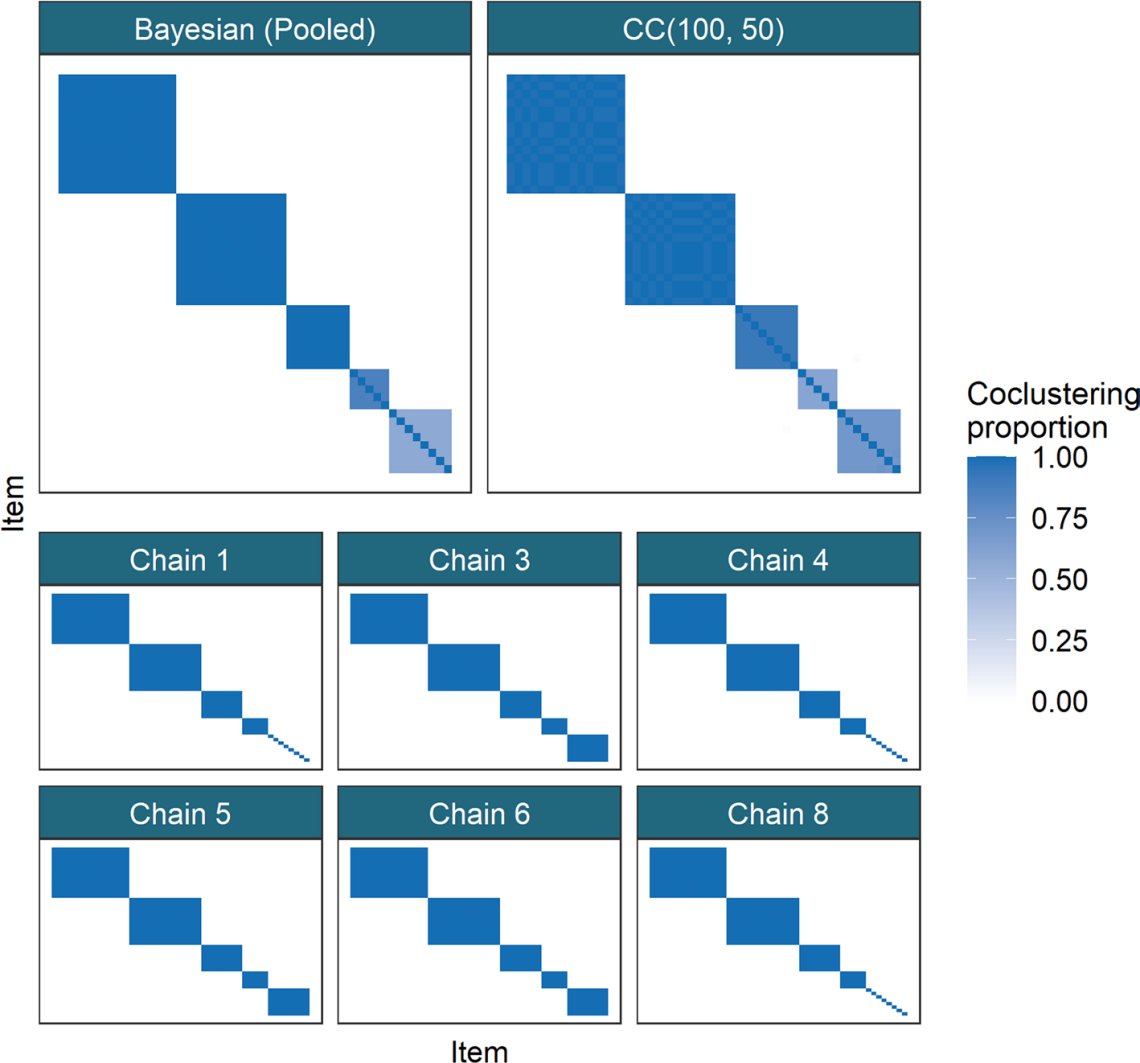
Comparison of similarity matrices from a dataset for the Small *N*, large *P* scenario. In each matrix, the (*i*, *j*)th entry is the proportion of clusterings for which the *i*th and *j*th items co-clustered for the method in question. In the first row the PSM of the pooled Bayesian samples is compared to the CM for CC(100, 50), with a common ordering of rows and columns in both heatmaps. In the following rows, 6 of the long chains that passed the tests of convergence are shown

**Fig. 4:**
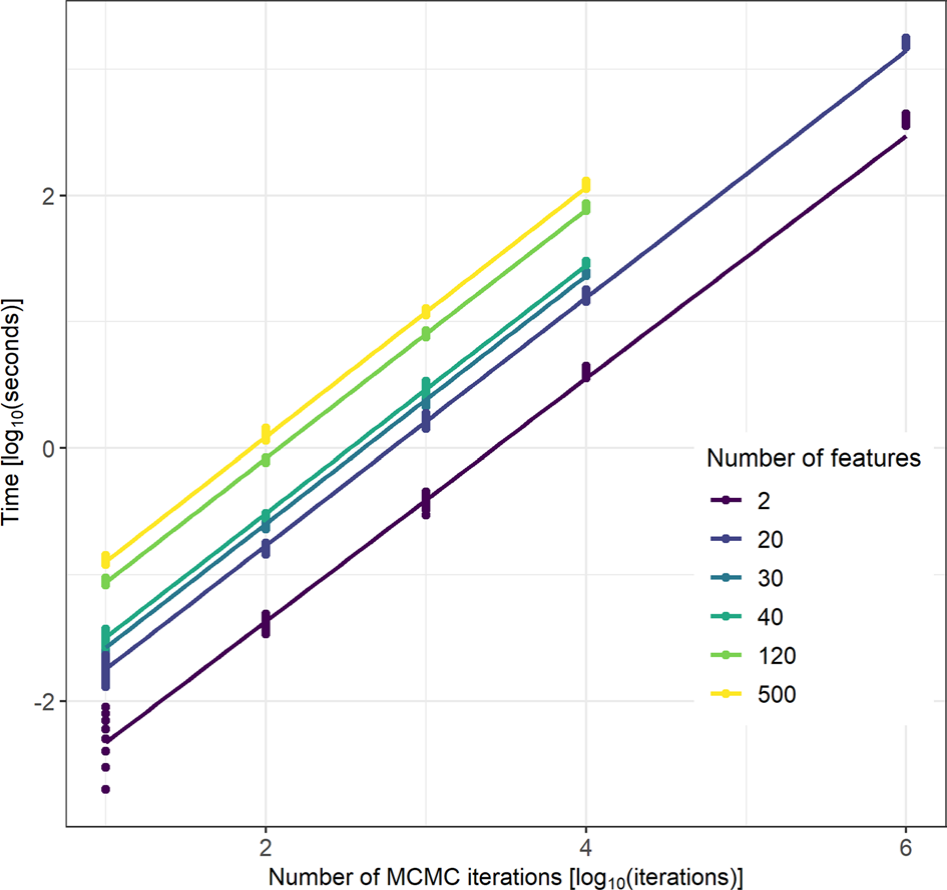
The time taken for different numbers of iterations of MCMC moves in $\log _{10}$(s). The relationship between chain length, *D*, and the time taken is linear (the slope is approximately 1 on the $\log _{10}$ scale), with a change of intercept for different dimensions. The runtime of each Markov chain was recorded using the terminal command time, measured in ms

For the PSMs from the individual chains, all entries are 0 or 1 (Fig. 3). This means only a single clustering is sampled within each chain, implying very little uncertainty in the partition. However, three different clustering solutions emerge across the chains, indicating that each individual chain is failing to explore the full support of the posterior distribution of the clustering. In general, while MCMC convergence theorems hold as the number of iterations tend to infinity, any finite chain might suffer in representing the full support of the posterior distribution, as we observe here. Moreover, the mixing of each chain can be poor as well (i.e. it may take a long time to reach the stationary distribution from an arbitrary initialisation). In our empirical study, we find that using many short runs provide similar point and interval estimates to running a small number of long chains (Fig. 3), while being computationally less expensive (Fig. 4), and hence more convenient for our applications.

Figure 4 shows that chain length is directly proportional to the time taken for the chain to run. This means that using an ensemble of shorter chains, as in consensus clustering, can offer large reductions in the time cost of analysis when a parallel environment is available compared to standard Bayesian inference. Even on a laptop of 8 cores running an ensemble of 1,000 chains of length 1,000 will require approximately half as much time as running 10 chains of length 100,000 due to parallelisation, and the potential benefits are far greater when using a large computing cluster.

Additional results for these and other simulations are in section 4.4 of the Additional file 1.

### Multi-omics analysis of the cell cycle in budding yeast

**Fig. 5:**
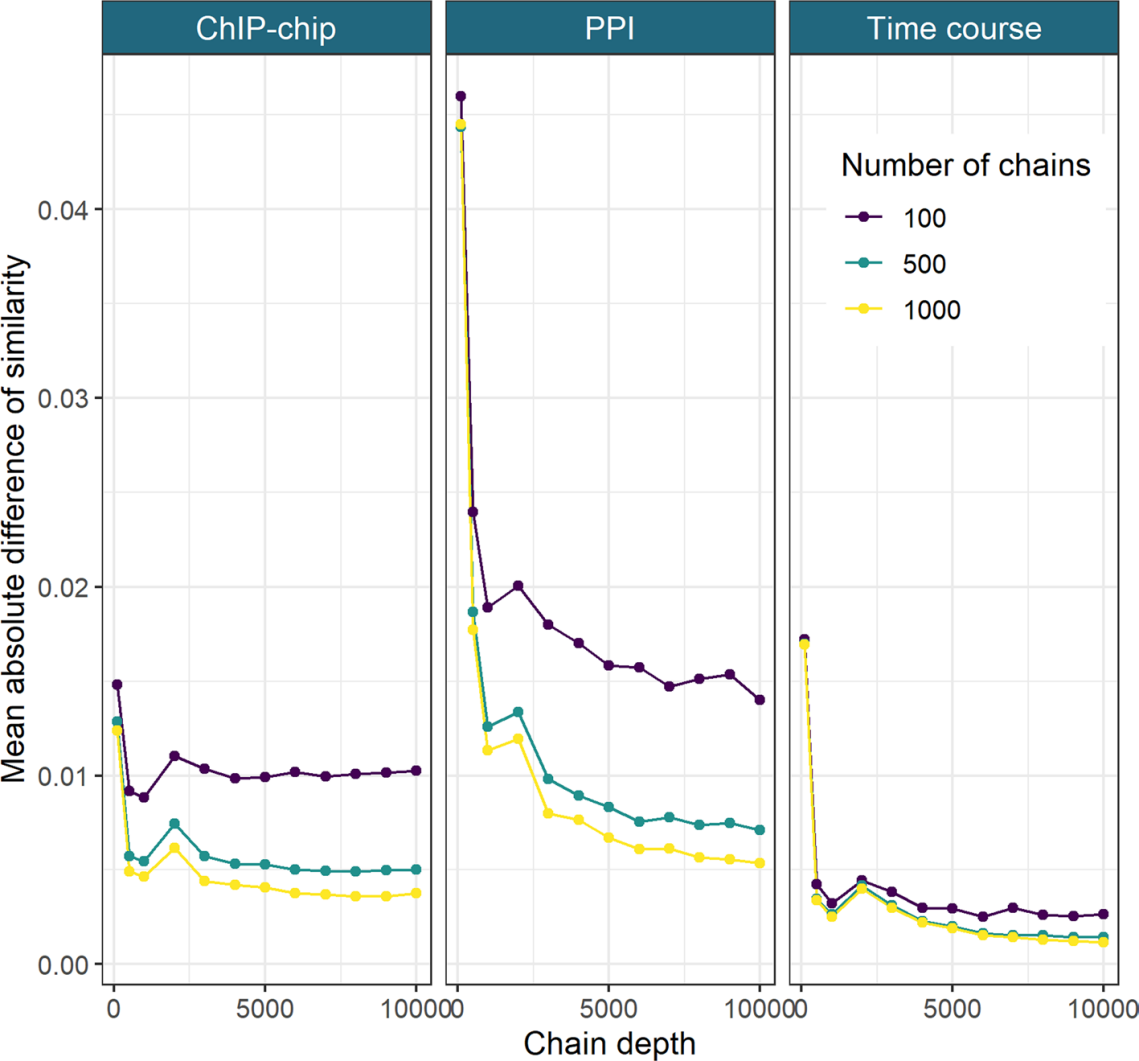
The mean absolute difference between the sequential Consensus matrices. For a set of chain lengths, $D'=\{d_1, \ldots , d_I\}$ and number of chains, $W'=\{w_1, \ldots , w_J\}$, we take the mean of the absolute difference between the consensus matrix for $(d_i, w_j)$ and $(d_{i-1}, w_{j})$ (here $D'=\{101, 501, 1001, 2001, \ldots , 10{,}001\}$ and $W'=\{100, 500, 1000\}$)

We use the stopping rule proposed in to determine our ensemble depth and width. In Fig. 5, we see that the change in the consensus matrices from increasing the ensemble depth and width is diminishing in keeping with results in the simulations. We see no strong improvement after *D* = 6000 and increasing the number of learners from 500 to 1,000 has small effect. We therefore use the largest ensemble available, a depth *D* = 10,001 and width *W* = 1000, believing this ensemble is stable (additional evidence in section 5.1 of the Additional file 1).

**Fig. 6:**
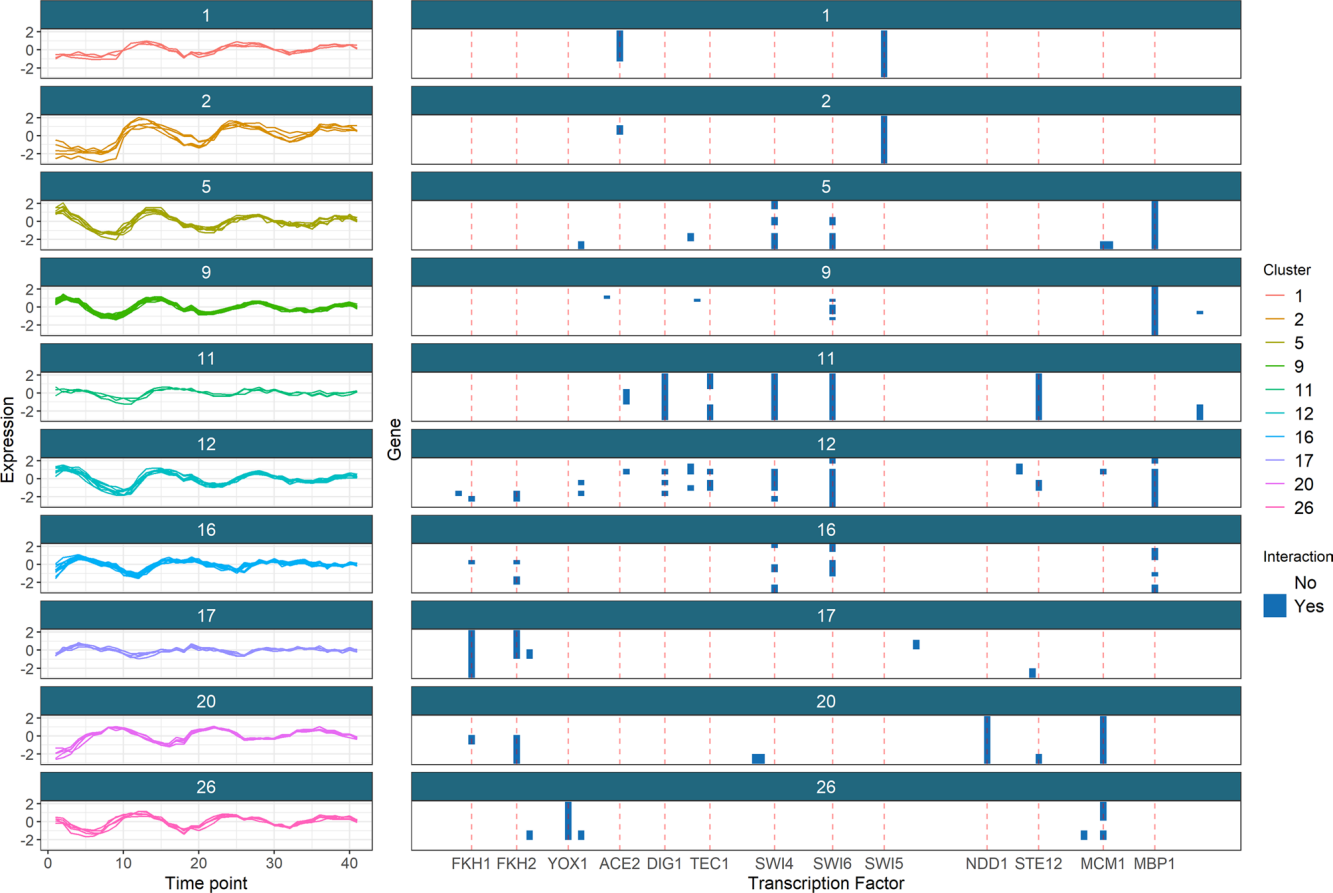
The gene clusters which tend to have a common label across the time course and ChIP-chip datasets, shown in these datasets. We include only the clusters with more than one member and more than half the members having some interactions in the ChIP-chip data. Red lines for the most common transcription factors are included

We focus upon the genes that tend to have the same cluster label across multiple datasets. More formally, we analyse the clustering structure among genes for which ${\hat{P}}(c_{nl} = c_{nm}) > 0.5$, where $c_{nl}$ denotes the cluster label of gene *n* in dataset *l*. In our analysis it is the signal shared across the time course and ChIP-chip datasets that is strongest, with 261 genes (nearly half of the genes present) in this pairing tending to have a common label, whereas only 56 genes have a common label across all three datasets. Thus, we focus upon this pairing of datasets in the results of the analysis performed using all three datasets. We show the gene expression and regulatory proteins of these genes separated by their cluster in Fig. 6. In Fig. 6, the clusters in the time series data have tight, unique signatures (having different periods, amplitudes, or both) and in the ChIP-chip data clusters are defined by a small number of well-studied transcription factors (**TFs**) (see table S2 for details of these TFs, many of which are well known to regulate cell cycle expression, [87].

As an example, we briefly analyse clusters 9 and 16 in greater depth. Cluster 9 has strong association with MBP1 and some interactions with SWI6, as can be seen in Fig. 6. The Mbp1-Swi6p complex, MBF, is associated with DNA replication [88]. The first time point, 0 min, in the time course data is at the START checkpoint, or the G1/S transition. The members of cluster 9 begin highly expressed at this point before quickly dropping in expression (in the first of the 3 cell cycles). This suggests that many transcripts are produced immediately in advance of S-phase, and thus are required for the first stages of DNA synthesis. These genes’ descriptions (found using org.Sc.sgd.db, [89] and shown in table S3) support this hypothesis, as many of the members are associated with DNA replication, repair and/or recombination. Additionally, *TOF1*, *MRC1* and *RAD53*, members of the replication checkpoint [90, 91] emerge in the cluster as do members of the cohesin complex. Cohesin is associated with sister chromatid cohesion which is established during the S-phase of the cell cycle [92] and also contributes to transcription regulation, DNA repair, chromosome condensation, homolog pairing [93], fitting the theme of cluster 9.

Cluster 16 appears to be a cluster of S-phase genes, consisting of *GAS3*, *NRM1* and *PDS1* and the genes encoding the histones H1, H2A, H2B, H3 and H4. Histones are the chief protein components of chromatin [94] and are important contributors to gene regulation [95]. They are known to peak in expression in S-phase [84], which matches the first peak of this cluster early in the time series. Of the other members, *NRM1* is a transcriptional co-repressor of MBF-regulated gene expression acting at the transition from G1 to S-phase [96, 97]. Pds1p binds to and inhibits the Esp1 class of sister separating proteins, preventing sister chromatids separation before M-phase [92, 98]. *GAS3*, is not well studied. It interacts with *SMT3* which regulates chromatid cohesion, chromosome segregation and DNA replication (among other things). Chromatid cohesion ensures the faithful segregation of chromosomes in mitosis and in both meiotic divisions [99] and is instantiated in S-phase [92]. These results, along with the very similar expression profile to the histone genes in the time course data, suggest that *GAS3* may be more directly involved in DNA replication or chromatid cohesion than is currently believed.

**Fig. 7:**
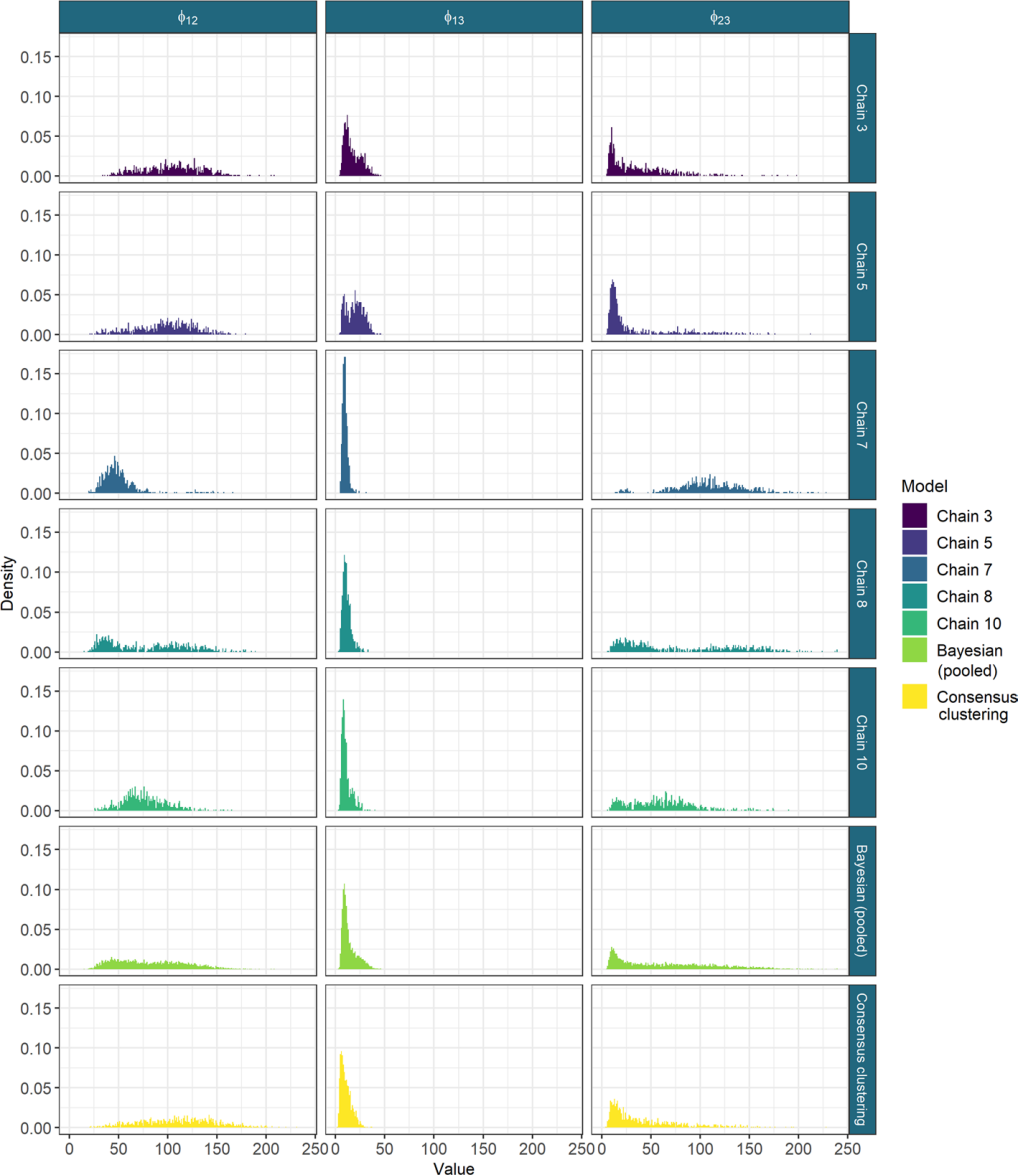
The sampled values for the $\phi$ parameters from the long chains, their pooled samples and the consensus using 1000 chains of depth 10,001. The long chains display a variety of behaviours. Across chains there is no clear consensus on the nature of the posterior distribution. The samples from any single chain are not particularly close to the behaviour of the pooled samples across all three parameters. It is the consensus clustering that most approaches this pooled behaviour

We attempt to perform a similar analysis using traditional Bayesian inference of MDI, but after 36 h of runtime there is no consistency or convergence across chains. We use the Geweke statistic and ${\hat{R}}$ to reduce to the five best behaved chains (none of which appear to be converged, see section 5.2 of the Additional file 1 for details). If we then compare the distribution of sampled values for the $\phi$ parameters for these long chains, the final ensemble used (D = 10,001, W = 1000) and the pooled samples from the 5 long chains, then we see that the distribution of the pooled samples from the long chains (which might be believed to sampling different parts of the posterior distribution) is closer in appearance to the distributions sampled by the consensus clustering than to any single chain (Fig. 7). Further disagreement between chains is shown in the Gene Ontology term over-representation analysis in section 5.3 of the Additional file 1.

## Discussion

Our proposed method has demonstrated good performance on simulation studies, uncovering the generating structure in many cases and performing comparably to Mclust and long chains in many scenarios. We saw that when the chains are sufficiently deep that the ensemble approximates Bayesian inference, as shown by the similarity between the PSMs and the CM in the 2D scenario where the individual chains do not become trapped in a single mode. We have shown cases where many short runs are computationally less expensive than one long chain and give meaningful point and interval estimates; estimates that are very similar to those from the limiting case of a Markov chain. Thus if individual chains are suffering from mixing problems or are too computationally expensive to run, consensus clustering may provide a viable option. We also showed that the ensemble of short chains is more robust to irrelevant features than Mclust.

We proposed a method of assessing ensemble stability and deciding upon ensemble size which we used when performing an integrative analysis of yeast cell cycle data using MDI, an extension of Bayesian mixture models that jointly models multiple datasets. We uncovered many genes with shared signal across several datasets and explored the meaning of some of the inferred clusters using data external to the analysis. We found biologically meaningful results as well as signal for possibly novel biology. We also showed that individual chains for the existing implementation of MDI do not converge in a practical length of time, having run 10 chains for 36 h with no consistent behaviour across chains. This means that Bayesian inference of the MDI model is not practical on this dataset with the software currently available.

However, consensus clustering does lose the theoretical framework of true Bayesian inference. We attempt to mitigate this with our assessment of stability in the ensemble, but this diagnosis is heuristic and subjective, and while there is empirical evidence for its success, it lacks the formal results for the tests of model convergence for Bayesian inference.

More generally, we have benchmarked the use of an ensemble of Bayesian mixture models, showing that this approach can infer meaningful clusterings and overcomes the problem of multi-modality in the likelihood surface even in high dimensions, thereby providing more stable clusterings than individual long chains that are prone to becoming trapped in individual modes. We also show that the ensemble can be significantly quicker to run. In our multi-omics study we have demonstrated that the method can be applied as a wrapper to more complex Bayesian clustering methods using existing implementations and that this provides meaningful results even when individual chains fail to converge. This enables greater application of complex Bayesian clustering methods without requiring re-implementation using more clever MCMC methods, a process that would involve a significant investment of human time.

We expect that researchers interested in applying some of the Bayesian integrative clustering models such as MDI and Clusternomics [32] will be enabled to do so, as consensus clustering overcomes some of the unwieldiness of existing implementations of these complex models. More generally, we expect that our method will be useful to researchers performing cluster analysis of high-dimensional data where the runtime of MCMC methods becomes too onerous and multi-modality is more likely to be present.

## Supplementary Information

**Additional file 1.** Additional relevant theory, background and results. This includes some more formal definitions, details of Bayesian mixture models and MDI, the general consensus clustering algorithm, additional simulations and the generating algorithm used, steps in assessing Bayesian model convergence in both the simulated datasets and yeast analysis, a table of the transcription factors that define the clustering in the ChIP-chip dataset, a table of the gene descriptions for some of the clusters that emerge across the timecourse and ChIP-chip datasets and Gene Ontology term over-representation analysis of the clusterings from the yeast datasets.

## Data Availability

The code and datasets supporting the conclusions of this article are available in the github repository, https://github.com/stcolema/ConsensusClusteringForBayesianMixtureModels.

## Abbreviations

ARI: Adjusted Rand Index
ChIP-chip: Chromatin immunoprecipitation followed by microarray hybridization
CM: Consensus matrix
MCMC: Markov chain Monte Carlo
MDI: Multiple dataset integration
PCA: Principal component analysis
PPI: Protein–protein interaction
PSM: Posterior similarity matrix
SSE: Sum of squared errors
TF: Transcription factor
