# Supplementary material for: Consensus clustering for Bayesian mixture models
Source: BMC Bioinformatics. 2022 Jul 21;23:290. doi: 10.1186/s12859-022-04830-8 (PMC9306175; doi:10.1186/s12859-022-04830-8)
Supplement: Supplementary file 1 — Additional file 1. Additional relevant theory, background and results. This includes some more formal definitions, details of Bayesian mixture models and MDI, the general consensus clustering algorithm, additional simulations and the generating algorithm used, steps in assessing Bayesian model convergence in both the simulated datasets and yeast analysis, a table of the transcription factors that define the clustering in the ChIP-chip dataset, a table of the gene descriptions for some of the clusters that emerge across the timecourse and ChIP-chip datasets and Gene Ontology term over-representation analysis of the clusterings from the yeast datasets. [file 12859_2022_4830_MOESM1_ESM.pdf]

Consensus clustering for Bayesian mixture models: Supplementary materials

Stephen Coleman, Paul DW Kirk and Chris Wallace

May 12, 2022

Abstract

Description of models used and analyses performed.

1 Definitions

Definition 1 (Coclustering matrix) *The* coclustering matrix *describes a clustering or partition in a binary matrix with the $(i, j)^{th}$ entry indicating if items i and j are allocated to the same cluster.*

Definition 2 (Consensus matrix) *Given W clusterings for a dataset of N items, $c_s = (c_{s1}, \ldots, c_{sN})$, the* consensus matrix *is a $N \times N$ matrix where the $(i, j)^{th}$ entry records the proportions of clusterings for which items i and j are allocated the same label. More formally, it is the matrix $\mathbb{C}$ such that*

$$\mathbb{C}(i, j) = \frac{1}{W} \sum_{s=1}^{W} \mathbf{I}(c_{si} = c_{sj}) \tag{1}$$

where $\mathbb{I}(\cdot)$ is the indicator function taking a value of 1 if the argument is true and 0 otherwise.

Definition 3 (Posterior similarity matrix) *A* consensus matrix *for which all the clusterings are generated from a converged Markov chain for some Bayesian clustering model. Sometimes abbreviated to* PSM.

Definition 4 (Partition *or* Clustering) *For a dataset of items $X = (x_1, \ldots, x_N)$, a* partition *or* clustering *is a set of disjoint sets covering X, normally indicated by a N-vector of integers indicating which set each item is associated with. Note that these labels only have meaning relative to each other, they are symbolic. Each set within the clustering is referred to as a* cluster.

2 The models

2.1 Individual dataset

In the simulations (see section 4) where individual datasets are modelled a *Bayesian mixture model* is used. We write the basic mixture model for independent items $X = (x_1, \dots, x_N)$ as

$$x_n \sim \sum_{k=1}^{K} \pi_k f(x_n|\theta_k) \qquad \text{independently for } n = 1, \dots, N \tag{2}$$

where $f(\cdot|\theta)$ is some family of densities parametrised by θ. A common choice is the Gaussian density function, with $\theta = (\mu, \sigma^2)$ (as in our simulation study). K, the number of subgroups in the population, $\{\theta_k\}_{k=1}^{K}$, the component parameters, and $\pi = (\pi_1, \dots, \pi_K)$, the component weights are the objects to be inferred. In the context of *clustering*, such a model arises due to the belief that the population from which the random sample under analysis has been drawn consists of K unknown groups proportional to π. In this setting it is natural to include a latent *allocation variable*, $c = (c_1, \dots, c_N)$, to indicate which group each item is drawn from, with each non-empty component of the mixture corresponds to a cluster. The model is

$$\begin{aligned} p(c_n = k) &= \pi_k \quad \text{for } k = 1, \dots, K, \\ x_n|c_n &\sim f(x_n|\theta_k) \quad \text{independently for } n = 1, \dots, N. \end{aligned} \tag{3}$$

The joint model can then be written

$$p(X, c, K, \pi, \theta) = p(X|c, \pi, K, \theta)p(\theta|c, \pi, K)p(c|\pi, K)p(\pi|K)p(K)$$

We assume conditional independence between certain parameters such that the model reduces to

$$p(X, c, \theta, \pi, K) = p(\pi|K)p(\theta|K)p(K) \prod_{n=1}^{N} p(x_n|c_n, \theta_{c_n})p(c_n|\pi, K). \tag{4}$$

Additional flexibility is provided by the inclusion of hyperparameters on the priors for π and θ, denoted α and η respectively. In our context where $\theta = (\mu, \sigma^2)$, we use

$$\sigma^2 \sim \Gamma^{-1}(a, b), \tag{5}$$

$$\mu \sim \mathcal{N}(\xi, \frac{1}{\lambda}\sigma^2), \tag{6}$$

$$\pi \sim \text{Dirichlet}(\alpha). \tag{7}$$

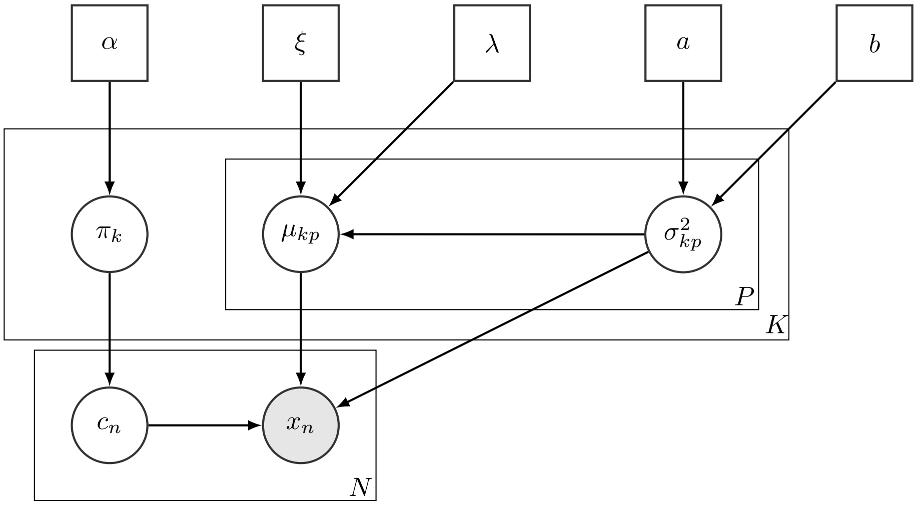

Figure S1: Directed acyclic graph for a mixture of Gaussians with independent features, as used in the simulation study.

The directed acyclic graph (**DAG**) for this model is shown in figure S1. The value of the hyperparameters we use are

$$\alpha = 1, \tag{8}$$
$$\xi = 0.0, \tag{9}$$
$$\lambda = 1.0, \tag{10}$$
$$a = 2.0, \tag{11}$$
$$b = 2.0. \tag{12}$$

2.2 Integrative clustering

We are interested in the use of consensus clustering for integrative methods. We used Multiple Dataset Integration (**MDI**, Kirk et al., 2012) as an example of a Bayesian integrative clustering method. MDI models dataset specific clusterings, in contrast to, for example, Clusternomics (Gabasova et al., 2017) in which a *global clustering* is inferred.

The defining aspect of MDI is the prior on the allocation of the nth item across the L datasets

$$p(c_{n1}, \ldots, c_{nL}) \propto \prod_{l=1}^{L} \pi_{c_{nl}l} \prod_{l=1}^{L-1} \prod_{m=l+1}^{L} (1 + \phi_{lm} \mathbb{I}(c_{nl} = c_{nm})) \text{ for } n = 1, \ldots, N. \tag{13}$$

ϕ_{lm} is the parameter defined by the similarity of the clusterings for the lth and m^{th} datasets and is also sampled in each iteration. As ϕ_{lm} increases more mass is placed on the common partition for these datasets. Conversely, in the limit $\phi_{lm} \to 0$ we have independent mixture models. In other words, MDI allows datasets with similar clustering of the items to inform the clustering in each other more strongly than the clustering for an unrelated dataset. The DAG for this model for three datasets is shown in figure S2.

3 Consensus clustering

Consensus clustering as described by Monti et al. (2003) applies W independent runs of the underlying clustering algorithm to perturbed versions of the dataset and combines the W final partitions in a *consensus matrix* which can be used to infer a final clustering. An outline of this is described in algorithm 1.

The consensus matrix is a symmetric matrix with the $(i, j)^{th}$ entry being the proportions of model runs for which the ith and j^{th} items are clustered together.

Data: $X = (x_1, \ldots, x_N)$
Input: A resampling scheme *Resample*
A clustering algorithm *Cluster*
Number of resampling iterations W
Set of cluster numbers to try $\mathcal{K} = \{K_1, \ldots, K_{max}\}$
Output: A predicted clustering, $\hat{Y}$
The predicted number of clusters present $\hat{K}$
begin
  for $K \in \mathcal{K}$ **do**
    `/* initialise an empty consensus matrix */`
    $\mathbf{M}^{(K)} \leftarrow \mathbf{0}_{N \times N}$;
    for $w = 1$ **to** W **do**
      $X^{(s)} \leftarrow Resample(X)$;
      `/* Cluster the peturbed dataset, represented in a coclustering matrix */`
      $\mathbf{B}^{(w)} \leftarrow Cluster(X^{(w)}, K)$;
      $\mathbf{M}^{(K)} \leftarrow \mathbf{M}^{(K)} + \mathbf{B}^{(s)}$;
    end
    $\mathbf{M}^{(K)} \leftarrow \frac{1}{W}\mathbf{M}^{(K)}$;
  end
  $\hat{K} \leftarrow$ best $K \in \mathcal{K}$ based upon all $\mathbf{M}^{(K)}$;
  $\hat{Y} \leftarrow$ partition X based upon $\mathbf{M}^{(\hat{K})}$;
end

Algorithm 1: Consensus clustering algorithm

To partition X based upon the consensus matrix, we use the R function `maxpear` (Fritsch, 2012). `maxpear` uses a sample average clustering, inferring

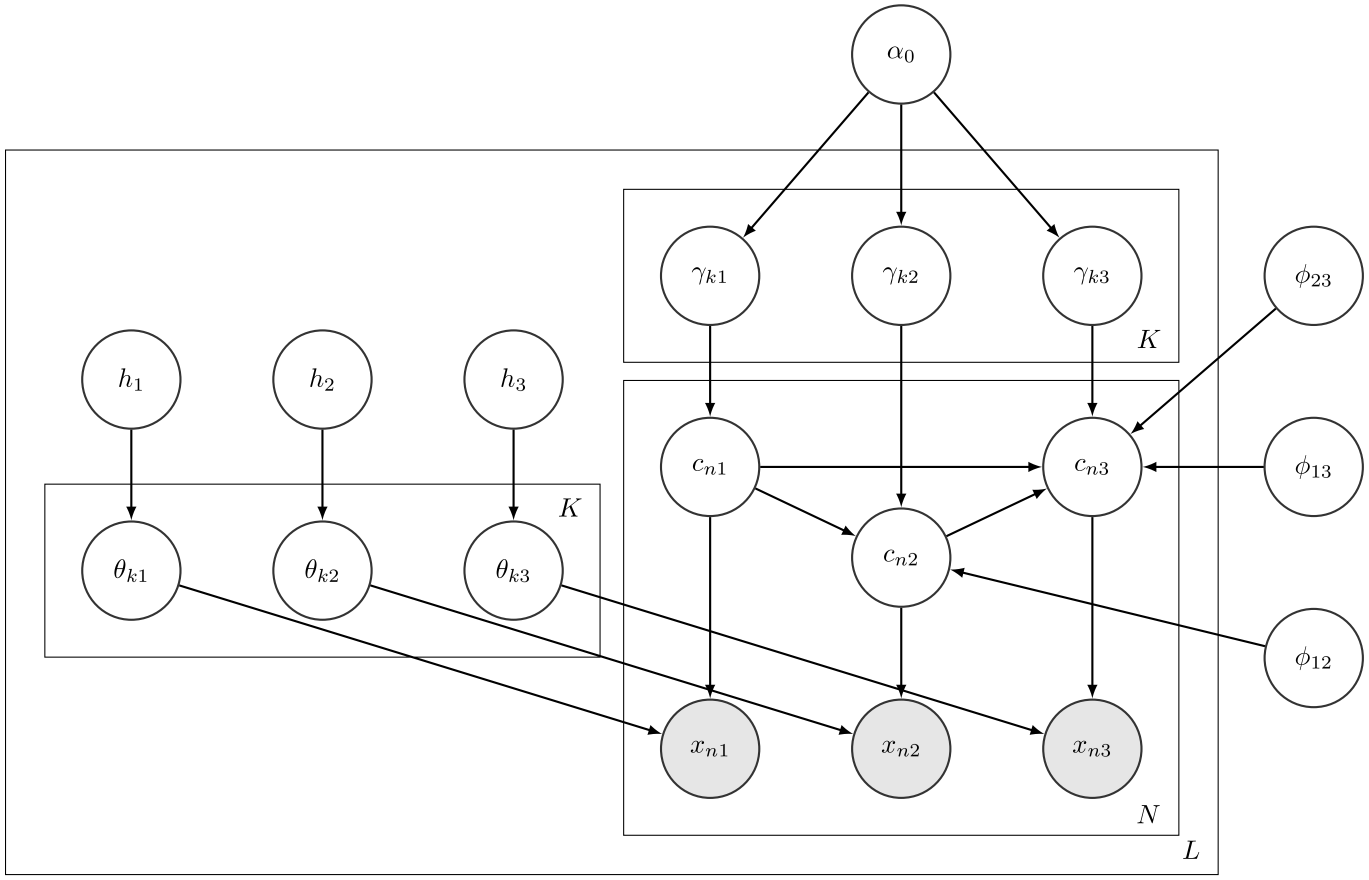

Figure S2: Directed acyclic graph for the Multiple Dataset Integration model for $L = 3$ datasets. h_l is the choice of hyperpriors for the lth dataset.

this by maximising the quantity

$$\frac{\sum_{i<j} \mathbb{I}(c_i^* = c_j^*) p_{ij} - \sum_{i<j} \mathbb{I}(c_i^* = c_j^*) \sum_{i<j} p_{ij} / \binom{N}{2}}{\frac{1}{2}\left[\sum_{i<j} \mathbb{I}(c_i^* = c_j^*) + \sum_{i<j} p_{ij}\right] - \sum_{i<j} \mathbb{I}(c_i^* = c_j^*) \sum_{i<j} p_{ij} / \binom{N}{2}} \tag{14}$$

where p_{ij} is the $(i,j)^{th}$ entry of the consensus matrix (Fritsch et al., 2009).

4 Simulated data

4.1 Scenario description

We defined 12 scenarios to simulate data within to test consensus clustering and to compare it to some alternative tools. Table S1 describes the parameters defining these scenarios and algorithm 2 describes how individual simulations were generated.

**Scenario**	N	P_s	P_n	K	$\Delta\mu$	σ^2	π
2D	100	2	0	5	3.0	1	$(\frac{1}{5}, \frac{1}{5}, \frac{1}{5}, \frac{1}{5}, \frac{1}{5})$
No structure	100	0	2	1	0.0	1	1
Base Case	200	20	0	5	1.0	1	$(\frac{1}{5}, \frac{1}{5}, \frac{1}{5}, \frac{1}{5}, \frac{1}{5})$
Large standard deviation	200	20	0	5	1.0	9	$(\frac{1}{5}, \frac{1}{5}, \frac{1}{5}, \frac{1}{5}, \frac{1}{5})$
Large standard deviation	200	20	0	5	1.0	25	$(\frac{1}{5}, \frac{1}{5}, \frac{1}{5}, \frac{1}{5}, \frac{1}{5})$
Irrelevant features	200	20	10	5	1.0	1	$(\frac{1}{5}, \frac{1}{5}, \frac{1}{5}, \frac{1}{5}, \frac{1}{5})$
Irrelevant features	200	20	20	5	1.0	1	$(\frac{1}{5}, \frac{1}{5}, \frac{1}{5}, \frac{1}{5}, \frac{1}{5})$
Irrelevant features	200	20	100	5	1.0	1	$(\frac{1}{5}, \frac{1}{5}, \frac{1}{5}, \frac{1}{5}, \frac{1}{5})$
Varying proportions	200	20	0	5	1.0	1	$(\frac{1}{2}, \frac{1}{4}, \frac{1}{8}, \frac{1}{16}, \frac{1}{16})$
Varying proportions	200	20	0	5	0.4	1	$(\frac{1}{2}, \frac{1}{4}, \frac{1}{8}, \frac{1}{16}, \frac{1}{16})$
Small N, large P	50	500	0	5	1.0	1	$(\frac{1}{5}, \frac{1}{5}, \frac{1}{5}, \frac{1}{5}, \frac{1}{5})$
Small N, large P	50	500	0	5	0.2	1	$(\frac{1}{5}, \frac{1}{5}, \frac{1}{5}, \frac{1}{5}, \frac{1}{5})$

Table S1: Parameters defining the simulation scenarios as used in generating data and labels.

We intend the scenarios to test different aspects of real data or to benchmark performance for comparison in the more challenging situations.

- *2D*: a low dimensional scenario within which we expected `Mclust` to perform well and the long chains to converge and explore the full support of the posterior distribution.
- *No structure*: we included this scenario to reassure fears that consensus clustering has a predilection to finding clusters where none exist (Şenbabaoğlu et al., 2014a,b).
- *Base case*: highly informative datasets within which we expected methods to find the true generating labels quite easily. We included this scenario to benchmark the others that are variations of this setting.

Algorithm: Simulation generation

Input: Distance between means Δ_μ
A common standard deviation σ^2
A number of clusters K
The number of items to generate in total N
The number of features to generate in total P
An indicator vector of feature relevance $\phi = (\phi_1, \ldots, \phi_P)$
The expected proportion of items in each cluster $\pi = (\pi_1, \ldots, \pi_K)$
A method for sampling x times from the array y, with weights π: $\mathit{Sample}(y, x, \pi)$
A method for permuting a vector x: $\mathit{Permute}(x)$
A method for generating a value from a univariate Gaussian distribution with mean μ and standard deviation σ^2: $\mathit{Gaussian}(\mu, \sigma^2)$

Output: A dataset, X
The generating cluster labels $c = (c_1, \ldots, c_N)$

begin
- `/* initialise the empty data matrix */`
- $X \leftarrow 0_{N \times P}$;
- `/* create a matrix of` K `means */`
- $\mu \leftarrow (\Delta_\mu, \ldots, K\Delta_\mu)$;
- `/* generate the allocation vector */`
- $c \leftarrow \mathit{Sample}(1:K, N, \pi)$;
- $\mathbf{M} \leftarrow \mathbf{0}_{N \times N}$;
- **for** $p = 1$ **to** P **do**
  - `/* Test if the feature is relevant, if relevant generate data from a mixture of univariate Gaussians, otherwise draw all items from the same distribution */`
  - **if** $\phi_p = 1$ **then**
    - $\nu \leftarrow \mathit{Permute}(\mu)$;
    - **for** $n = 1$ **to** N **do**
      - $X(n, p) \leftarrow \mathit{Gaussian}(\nu_{c_n}, \sigma^2)$
    - **end**
  - **end**
  - **if** $\phi_p = 0$ **then**
    - **for** $n = 1$ **to** N **do**
      - $X(n, p) \leftarrow \mathit{Gaussian}(0, \sigma^2)$
    - **end**
  - **end**
- **end**
- `/* Mean centre and scale the data */`
- $X \leftarrow Normalise(X)$

end

Algorithm 2: Data generation for a mixture of Gaussian with independent features. This algorithm is implemented in the `generateSimulationDataset` function from the `mdiHelpR` package available at `www.github.com/stcolema/mdiHelpR`.

- *Large standard deviation*: these two scenarios investigated the degree of distinction required between clusters for the methods to uncover their structure.
- *Irrelevant features*: we included these scenarios to investigate how robust the methods are to irrelevant features.
- *Varying proportions*: these scenarios investigated how well each method uncovers clusters when the clusters have significantly different membership counts.
- *Small N, large P*: an investigation of behaviour when the number of features is far greater than the number of items.

4.2 `Mclust`

We called `Mclust` using the default settings and a range of inputs for the choice of K. We used $K = (2, \ldots, \min(\frac{N}{2}, 50))$ to mirror the choice of $K_{max} = 50$ used for the overfitted mixture models (the default in the software we used), with the bound of $\frac{N}{2}$ to avoid fitting 50 clusters in the *Small N, large P* scenario where $N = 50 = K_{max}$. In the *No structure* scenarios we extended to range to $K = (1 \ldots, 50)$ to include the correct structure as an option. The model choice was performed using the Bayesian Information Criterion (Schwarz et al., 1978, as implemented in `Mclust`). `Mclust` tries different covariance matrices and thus the model choice is not just between different values of K.

4.3 Bayesian analysis

We use the implementation of Bayesian mixture models in C++ provided by Mason et al. (2016). Rather than directly using a Dirichlet process (Ferguson, 1973) to infer the number of clusters or a mixture that grows and shrinks (Richardson and Green, 1997), this implementation follows the logic of Rousseau and Mengersen (2011) and Van Havre et al. (2015) using an overfitted mixture model to approximate a Dirichlet process. In overfitted mixture models, the number of components, K_{max}, included in the model is set to number far larger than the true number of clusters, K, present.

For each simulation we ran 10 chains for 1 million iterations, keeping every thousandth sample. We discarded the first 10,000 iterations to account for burn-in bias, leaving 990 samples per chain. To check if the chains were converged we used

- the Geweke convergence diagnostic (Geweke et al., 1991) to investigate within-chain stationarity, and
- the potential scale reduction factor ($\hat{R}$, Gelman et al., 1992) and the Vats-Knudson extension (*stable* $\hat{R}$, Vats and Knudson, 2018) to check across-chain convergence.

The Geweke convergence diagnostic is a standard Z-score; it compares the sample mean of two sets of samples (in this case buckets of samples from the first half of the samples to the sample mean of the entire second half of samples). It is calculated under the assumption that the two parts of the chain are asymptotically independent and if this assumption holds (i.e. the chain is sampling the same distribution in both samples) than the scores are expected to be standard normally distributed. If a chain's Geweke convergence diagnostic passed a Shapiro-Wilks test for normality (Shapiro and Wilk, 1965) (based upon a threshold of 0.05), we considered it to have achieved stationarity and included it in the model performance analysis.

$\hat{R}$ is expected to approach 1.0 if the set of chains are converged. Low $\hat{R}$ is not sufficient in itself to claim chain convergence, but values above 1.1 are clear evidence for a lack of convergence (Gelman et al., 2013). Vats and Knudson (2018) show that this threshold is significantly too high (1.01 being a better choice) and propose extensions to $\hat{R}$ that enable a more formal rule for a threshold. We use their method as implemented in the R package `stableGR` (Knudson and Vats, 2020) as the final check of convergence. An example of the $\hat{R}$ series across the 100 simulations for a scenario where chains are well-behaved is shown in figure S3.

We focused upon stationarity of the continuous variables as assesing convergence of the allocation labels is difficult due to label-switching. In our simulations the only recorded continuous variable is the concentration parameter of the Dirichlet distribution for the component weights.

We pooled the samples from the stationary chains and used these to form a PSM. This and the point estimate clustering found by applying the R function `maxpear`. In Bayesian inference, `maxpear` attempts to find the clustering that maximises the Adjusted Rand Index to the true clustering by using an approximation of the expected clustering under the posterior, $\mathbb{E}(c|X)$, believing that this converges to the true clustering. A sample average clustering is used to approximate the expected clustering. This is estimated from the PSM by maximising

$$\frac{\sum_{i<j}\mathbb{I}(c_i^* = c_j^*)p_{ij} - \sum_{i<j}\mathbb{I}(c_i^* = c_j^*)\sum_{i<j}p_{ij}/\binom{N}{2}}{\frac{1}{2}\left[\sum_{i<j}\mathbb{I}(c_i^* = c_j^*) + \sum_{i<j}p_{ij}\right] - \sum_{i<j}\mathbb{I}(c_i^* = c_j^*)\sum_{i<j}p_{ij}/\binom{N}{2}} \tag{15}$$

where p_{ij} is the $(i,j)^{th}$ entry of the PSM (Fritsch et al., 2009). When the chain has converged this maximises the posterior expected ARI to the true clustering.

There are three possibilities to consider the decision to pool the samples across chains under:

- The chains are converged and agree upon the distribution sampled (see figure S4 for an example).
- The chains are not in agreement upon the partition sampled, becoming trapped in different modes. However, a mode does dominate being the mode present in a majority of chains (see figure S5 for an example of this behaviour).

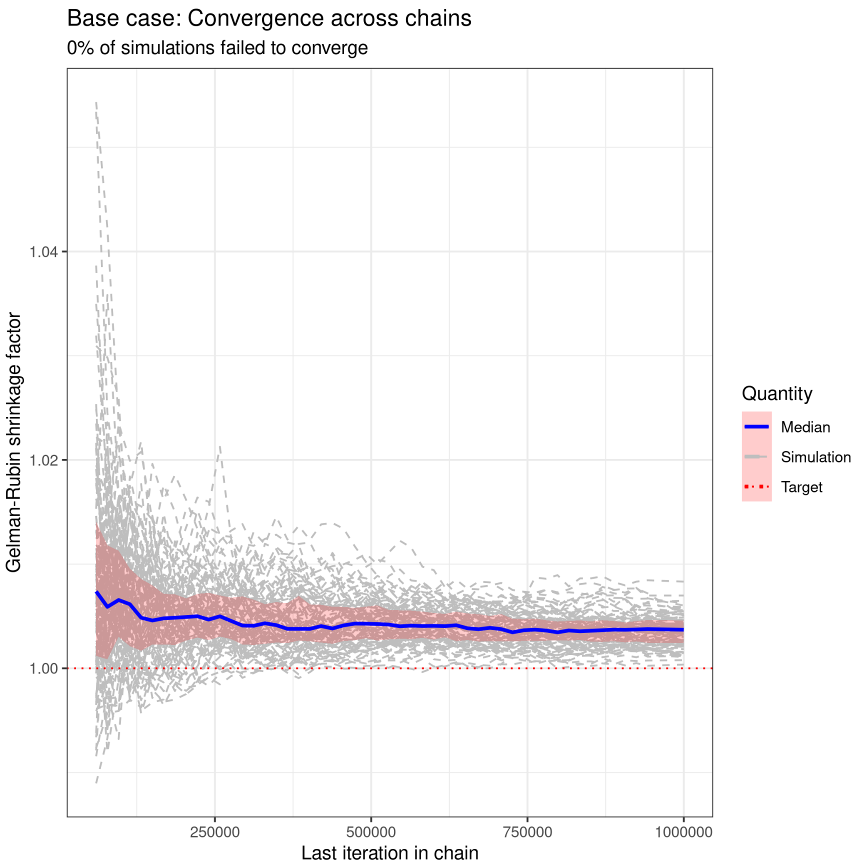

Figure S3: The $\hat{R}$ values for each simulation (in dotted grey), the median value and the interquartile range across simulations. One can see that $\hat{R}$ approaches 1.0, being below 1.01 for every simulation by the end of the chains. The "0% of simulations failed to converge" is a statement based upon the percentage of simulations which passed the test of stable $\hat{R}$.

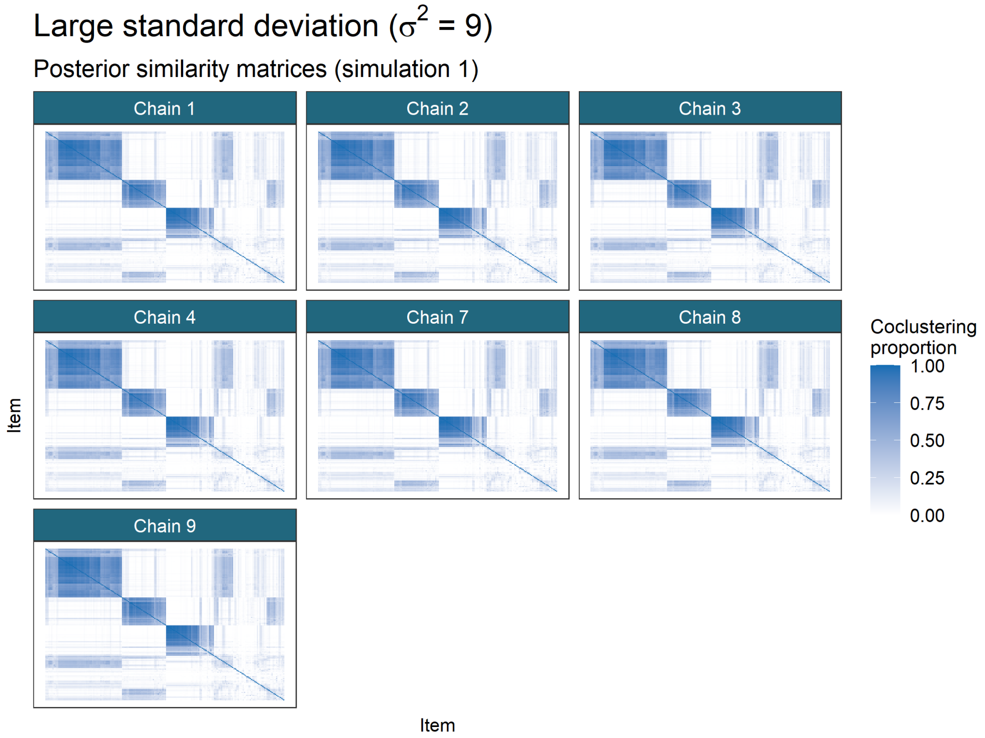

Figure S4: Posterior similarity matrices for the simulation generated using a random seed set to 1 for the first large standard deviation scenario from table S1. This is an example of all stationary chains agreeing in a simulation (and thus pooling of samples is no different to using any choice of chain for the performance analysis). Ordering of rows and columns is defined by hierarchical clustering of the first matrix in the series, in this case that from Chain 1.

- The chains are not in agreement and no one mode dominates among chains (see figure S6 for an example of this behaviour).

In the first case pooling has no effect upon the predicted clustering compared to using any one chain. In the second case it feels natural that one would use the mode that dominates. Pooling the samples effectively does this for the predictive performance of the method as the mode with the greatest number of samples across the chains dominates; however, the uncertainty for this mode is increased. In the third case the analysis is non-trivial and further thought, chains and samples would be required. In our simulations this case only arises in the most pathological form in the second *Large N, small P* scenario, where each chain remains trapped in the initial partition. The clustering inferred from any chain is not meaningful being a random clustering; thus the clustering predicted by pooling the PSMs is no more or less relevant as it too is random.

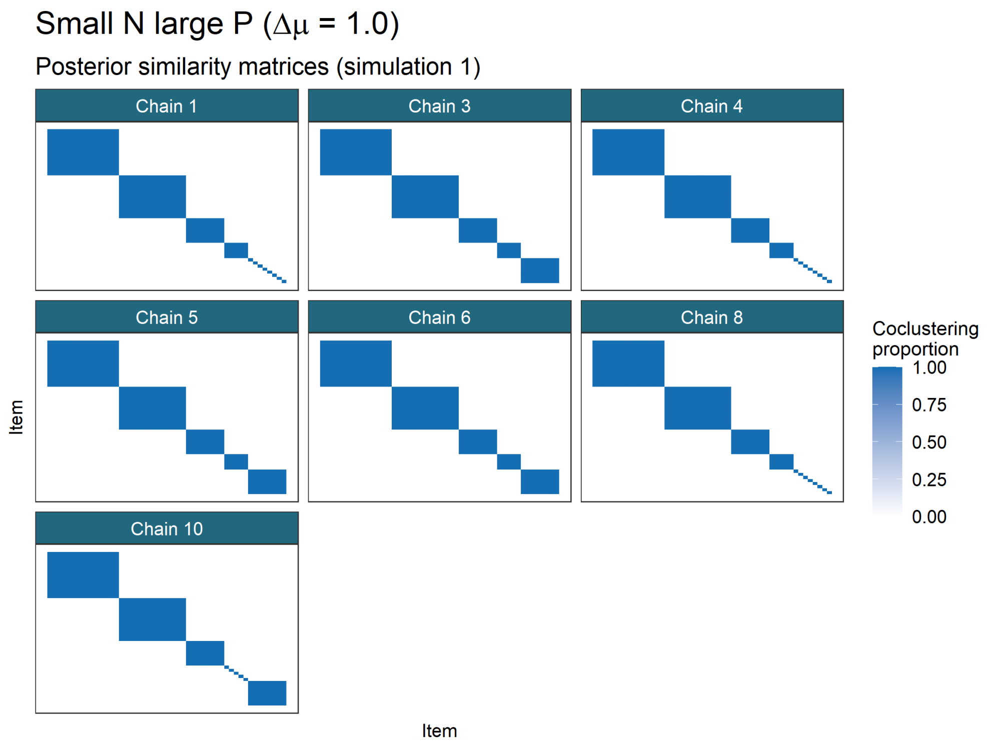

Figure S5: Posterior similarity matrices for the simulation generated using a random seed set to 1 for the first small N, large P scenario from table S1. This is an example of different chains becoming trapped in different modes, but one mode (which does represent the generating structure well) is dominant, being fully present in 3 of the 6 chains, with the two other modes present having significant overlap. Ordering of rows and columns is defined by hierarchical clustering of the first matrix in the series, in this case that from Chain 1.

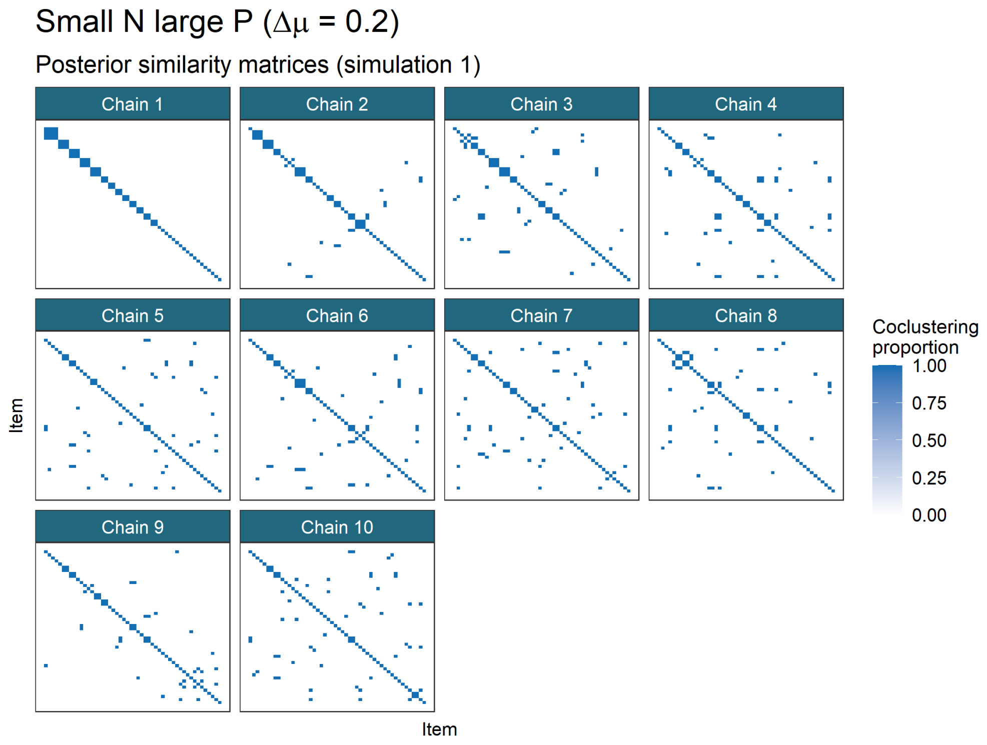

Figure S6: Posterior similarity matrices for the simulation generated using a random seed set to 1 for the second small N, large P scenario from table S1. This is an example of different chains becoming trapped in different modes with no mode being dominant. In this scenario each chain remains trapped in initialisation. Ordering of rows and columns is defined by hierarchical clustering of the first matrix in the series, in this case that from Chain 1.

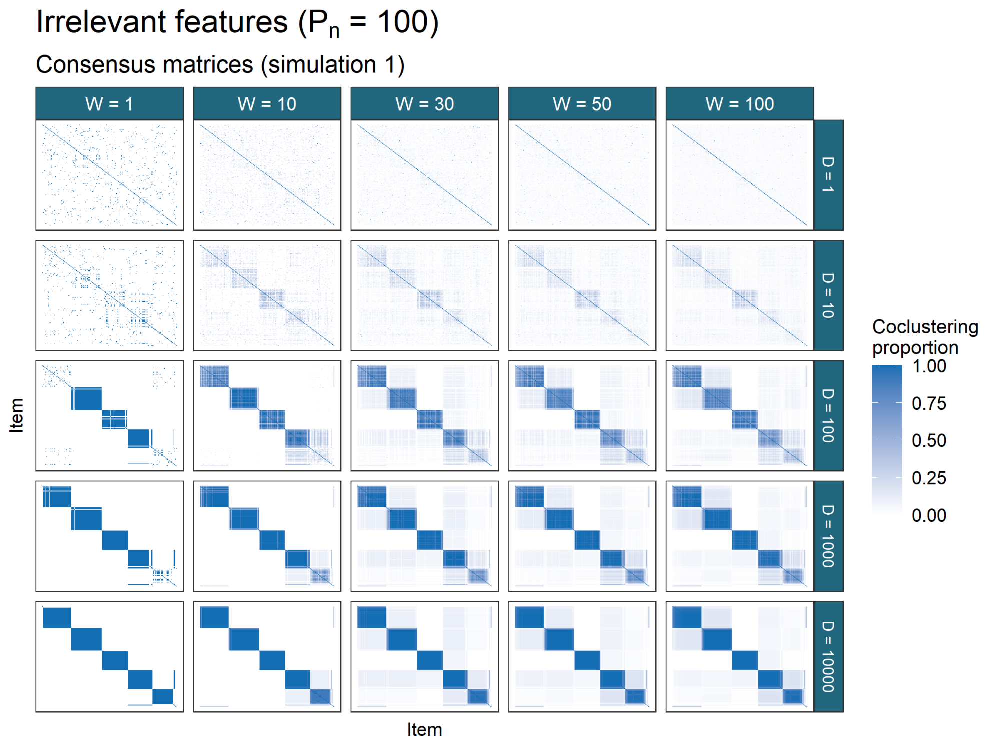

Figure S7: Consensus matrices for the simulation generated using a random seed set to 1 for the third irrelevant features scenario from table S1. D is the individual chain length and W is the number of chains used. In this example there are several modes present (as seen in the entries with values between 0 and 1) but one mode is clearly dominant (the 5 dark squares along the diagonal which correspond closely to the generating labels).

4.4 Consensus clustering analysis

We investigated a range of ensembles, using all combinations of chain depth, $D = \{1, 10, 100, 1000, 10000\}$, and the number of chains, $W = \{1, 10, 30, 50, 100\}$. This gave a total of 25 different ensembles. A consensus matrix was constructed from the samples generated by each ensemble by finding the proportion of samples within which any pair of items are coclustered. An example of the Consensus matrices for each ensemble in a given simulation is shown in figure S7.

4.5 Model performance

The different models (Bayesian (pooled), `Mclust` and the 25 consensus clustering ensembles) were compared under their ability to uncover the generating clustering.

In figure S11 the ARI between the generating labels and the point estimate clustering from each method is shown. For two partitions c_1, c_2,

- $ARI(c_1, c_2) = 1.0$: a perfect match between the two partitions,
- $ARI(c_1, c_2) = 0.0$: c_1 is no more similar to c_2 than is expected for a random partition of the data.

In several scenarios `Mclust` performs the best under this metric (e.g. in the scenarios *2D*, *Small N, large P ($\Delta\mu = 0.2$)*). However when the number of irrelevant features is large `Mclust` performs less well (see *Irrelevant features ($P_n = 20$)* and *($P_n = 100$)*) than the other methods. In the scenario that $P_n = 100$ failing to find structure is not inherently wrong as a majority of the features suggest that there are no subpopulations.

For the ensembles there are two parameters changing between each model, the iteration used to provide the clustering in the ensemble, D, and the number of chains (and hence samples) used, W. In many of the scenarios we find that the benefit of increasing D stabilises by approximately $D = 10$. We believe that in a low-dimensional dataset (such as *2D*), or a highly informative dataset (such as *Base case* or any of the higher dimensional scenarios with no irrelevant features where $\frac{\Delta\mu}{\sigma^2} \geq 1$) the chains quickly find a "sensible" partition of the data and thus increasing the depth within the chain does not increase the probability that any partition sampled will be closer to the generating partition. For example in figure S11 in the *Small N, large P* case, the distribution of the ARI across the ensembles for which $D \geq 10$ and $W = 1$ is nearly identical; this suggests that the chain is sampling a very similar partition again and again for 9,990 iterations (and possibly beyond based upon the PSMs shown in figure S5) and it is through adding more chains rather than using particularly long chains that we improve the ability to uncover the generating structure.

We also notice that even if the behaviour has not stabilised for D that the ensemble can uncover meaningful structure. The ARI for the ensembles of short chains can be quite high (as is the case in many of the scenarios). The behaviour of the consensus matrices also shows that low D is not a disqualifier from meaningful inference even if longer chains would be ideal, a result that might be useful in real applications with large datasets and complex models. Consider the consensus matrices in figure S7, it can be seen that the behaviour has not stabilised before $D = 10000$ (and possibly there is still some benefit in increasing D beyond this value), but the structure being uncovered when there is a sufficient number of chains and D is small does correspond to the structure uncovered in the largest and deepest ensemble. We believe that the order in which components merge and items are co-clustered varies depending on initialisation, and thus if the chain is not sufficiently deep that all of the final mergings have occured that a sufficiently large ensemble can still perform meaningful inference of the subpopulation structure despite the poor performance of any individual model. Even though each learner probably has too many clusters for small D the consensus among them will have less if the individual learners have low correlation between their partitions (something we might expect if the chains

are stopped very early). This is why the entries of the consensus matrix for $D = 100$ and $W = 100$ in figure S7 are more pale than in deeper ensembles; very few items correctly (possibly none) cocluster in every partition, it is only in observing the consensus that the global structure of interest emerges. Thus if there is some limit to the length of chains available for an analysis (e.g. computational or temporal constraints) than the inference obtained from the shorter chains can still be meaningful, with the caveat that the point clustering might have more clusters than the same analysis with longer chains would provide. Additional post-hoc merging of some clusters might be necessary in this case.

In contrast, when the dataset is sparse or contains many irrelevant features, we believe that deeper chains are required to reach this steady-state sampling where no single sample is expected to be better than any other (see the *Irrelevant features ($P_n = 100$)* facet of figure S11).

In some scenarios no method is successful in uncovering the generating labels. In the *Large standard deviation ($\sigma^2 = 25$)* and *Small N, large P ($\Delta\mu = 0.2$)* this is due to the lack of signal - the clusters overlap so significantly that it is not possible for any of these methods to uncover much of the generating structure. In the *No structure* case it is different (although `Mclust` does perform well here). In this case all items are generated from a common distributions. For the Bayesian chains and the ensembles, a clustering of singletons is predicted; each item is allocated a unique label (see figures S8 and S9). While failing to perform well under the ARI, this is a sensible result. Rather than indicating (as we did with the shared label) that no item is particularly distinct from the others and thus all share a common label, this clustering of singletons states that no item is more similar to any other and thus no two items should cluster together. It is an alternative statement of the same result, i.e. that there is no evidence for subpopulation structure. We consider this evidence that an ensemble of Bayesian mixture models is not as susceptible to predicting labels than an ensemble based upon K-means clustering as in Şenbabaoğlu et al. (2014a,b).

Increasing W is also required when the dimensionality of the dataset is large. In this case it is due to individual chains exploring only a single mode (as can be seen in figure S5 where each chain appears to sample only a single partition). In this example where each sample is a partition that appears to be a mode in the posterior distribution of the allocation vector from very early in the chain (based upon the stable performance for $D \geq 10$), increasing W allows each chain to "vote" on which mode is the global mode, as we believe that the mode that attracts the most chains is the global mode (although in real datasets the number of chains required might be greater than in our simulations). An example of this behaviour may be seen in figure S10.

In figure S11, limiting behaviour for increases of W and D can be seen for the ensemble. For most simulations there is no change in performance for greater choices of W and D after some stabilising values.

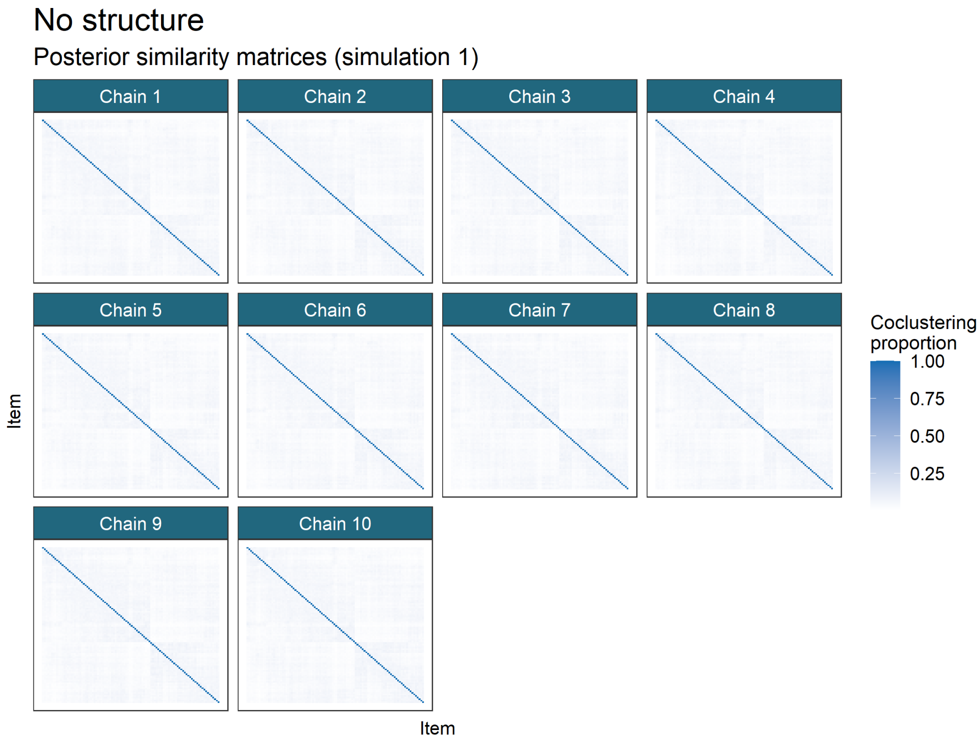

Figure S8: Posterior similarity matrices for simulation 1 of the *No structure* scenario. Each item is allocated to a singleton.

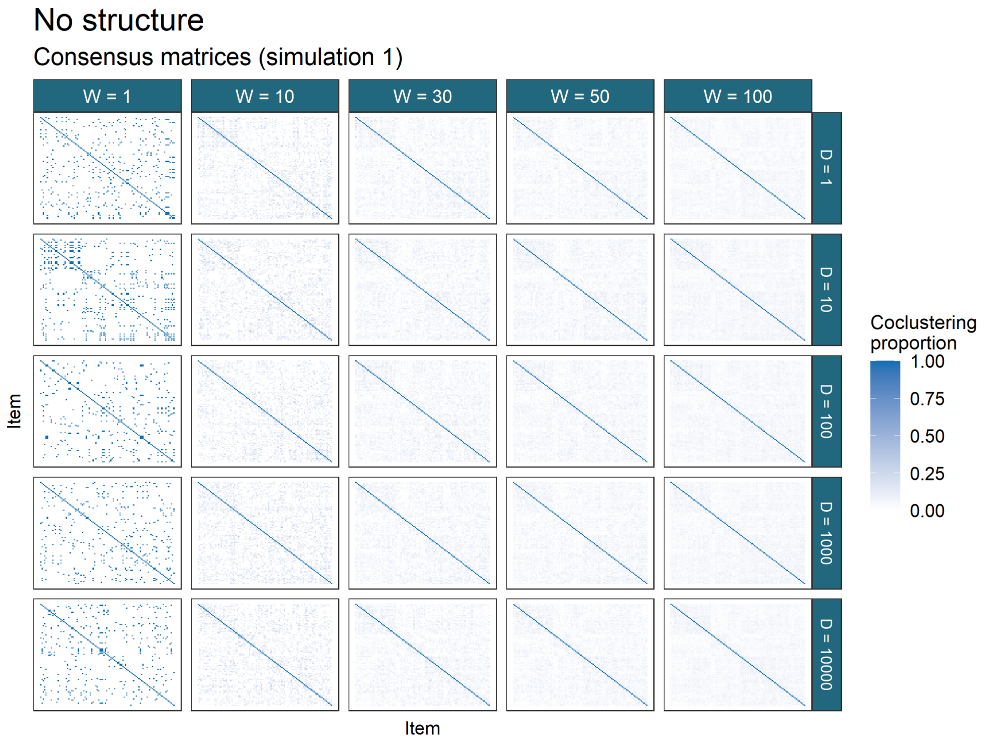

Figure S9: Consensus matrices for simulation 1 of the *No structure* scenario. Each item is allocated to a singleton in many of the Consensus matrices.

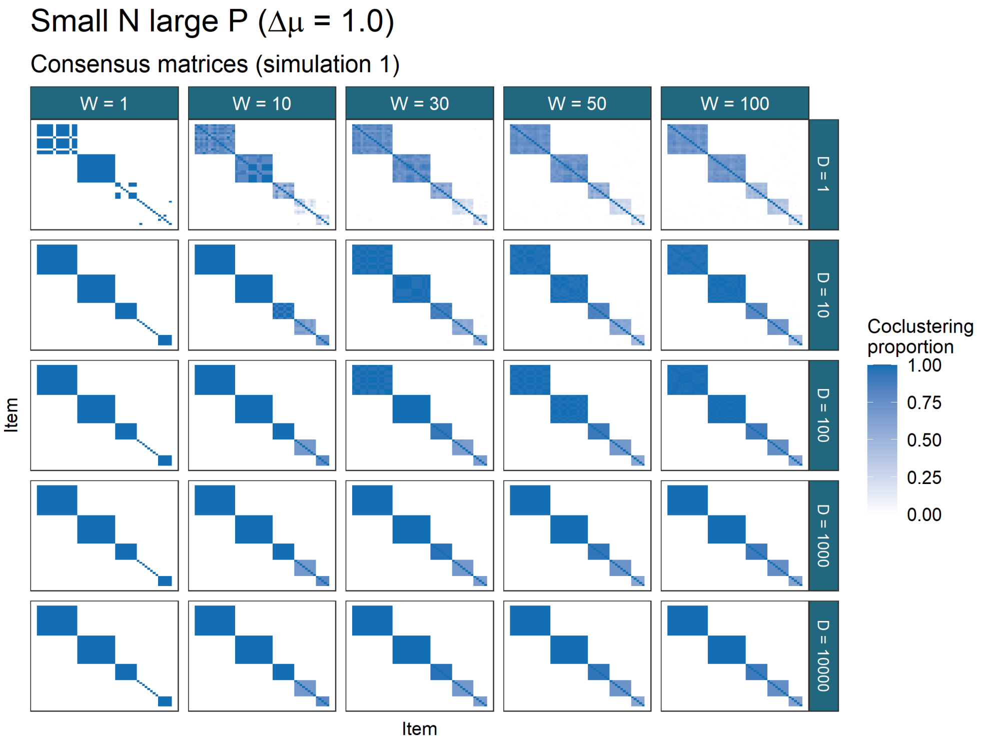

Figure S10: Consensus matrices for simulation 1 of the first *Large* N*, small* P scenario. One can see that by iteration ten the sample being drawn is from the mode (for $W = 1$, $D = 10$), and that an ensemble of chains does find structure that recalls the generating labels (see figure S11, the ARI for $CC(10, s)$ is 1.0 for $s > 1$, meaning that the true labels perfectly align with those predicted by the consensus matrix).

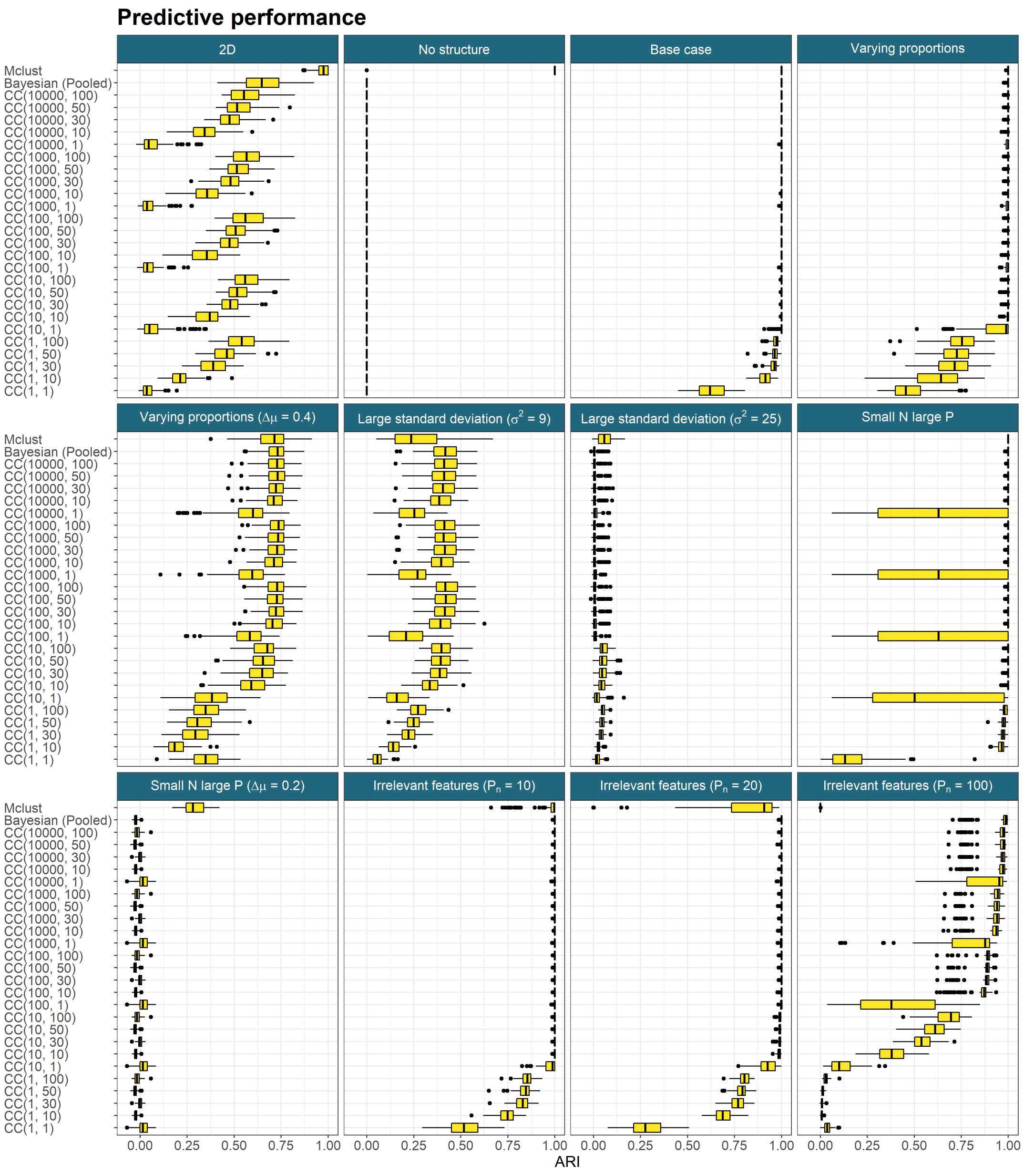

Figure S11: Predictive performance across all simulations. $CC(D,W)$ denotes consensus clustering using the Dth sample from W different chains. In the cases where the generating structure is not exactly found, increasing D and W sees some improvement in the ARI between the truth and the predicted clusterings before some limiting behaviour emerges and and further increase appears to have no change in the performance.

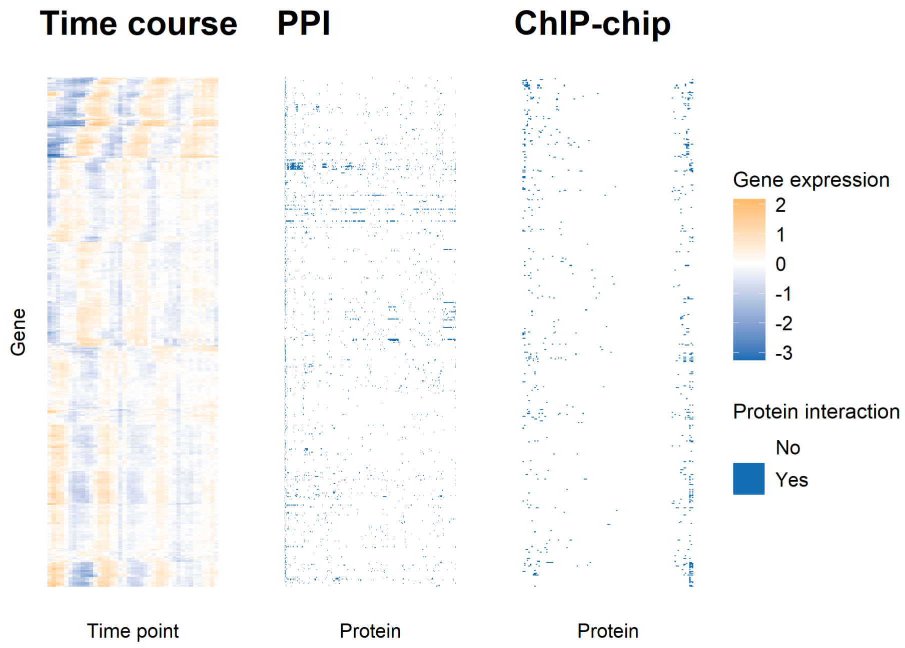

Figure S12: Heatmap of the yeast datasets. Each plot has a common row order corresponding to the gene products being clustered. This order was decided by a hierarchical clustering of the rows of the time course expression matrix. The time course data is associated with the "Gene expression" legend and the ChIP-chip and PPI data with "Protein interaction" legend.

5 Multi-omics analysis of the cell cycle in budding yeast

We chose our three datasets (shown in figure S12) to perform an integrative analysis as many of the protein encoding genes in the mitotic cell cycle have well studied genomic binding sites with mapped transcription factors (**TFs**) that control phase-specific expression (Cho et al., 1998; Spellman et al., 1998); thus the inclusion of the ChIP-chip data means that the clusters that align across the datasets should include well studied regulatory proteins and thus be of biological interest. If a cluster of genes are similarly expressed in the time course , share associated regulatory protein in the ChIP-chip and are associated with common protein complexes in the PPI data, than this implies a gene set with strong biological significance.

In contrast, if we cluster the time course dataset alone, any clusters that we find are defined by correlation across time. This might be assumed to be driven by shared regulatory mechanisms, but other sources of structure might

be encouraging this, even experimental error. However, if a cluster aligns across both the time course dataset and the ChIP-chip dataset we can be more certain that these genes are part of some regulatory network; if this cluster also emerges in the PPI dataset we might believe that the genes are co-regulated as part of the formation of some protein complex. Furthermore, this integrative aspect means that clusters that might merge in the time course dataset due to similar periodicity in a standalone analysis might remain separate due to different associated transcription factors in the ChIP-chip dataset.

Thus we performed an integrative analysis using MDI to avoid agressive assumptions about either the biology defining any clusters and modelling assumptions about the latent structure.

We expect that the complexity of this data and model means that the time required for convergence of the MCMC algorithm would be very large. We avoid this problem by using consensus clustering of MDI, instead basing our final ensemble choice on the stopping rule described in the main paper.

The datasets were modelled using a mixture of Gaussian processes in the time course dataset and Multinomial distributions in the ChIP-chip and PPI datasets. To ensure that our mixture model is initially overfitted we set $K_{max} = 275 \approx \frac{N}{2}$, and following from this the point estimate was inferred from the consensus matrix using `maxpear` as in the simulated data except we set `k.max = 275`.

5.1 Consensus clustering analysis

We include the consensus matrices for each dataset for a range of ensembles for further evidence that the ensemble was stable for the $10,000^{th}$ iteration from 1,000 chains in figures S13, S14 and S15. In these figures, there is no strong change between the consensus matrices for $D = 5001$ and $D = 10001$.

We wish to identify groups of genes that tend to be grouped together in multiple datasets. We focus upon the genes that tend to have the same cluster label in multiple datasets, those which have a common label across some set of datasets in more than half of the observed clusterings, or $\hat{P}(c_{nl} = c_{nm}) > 0.5$, where c_{nl} denotes the cluster label of gene n in dataset l. This based upon the the concept of *fused genes* proposed by Savage et al. (2010) and used by Kirk et al. (2012), but to avoid confusion due to other possible ideas of fused genes (e.g. those that contribute to a common protein complex, the behaviour of TFs upon a gene) we avoid this term. These genes with common clustering across datasets are those most affected by the integrative aspect of the analysis and therefore we focus upon these in the our cluster analysis. In our case we have the possible sets of:

- {Time course}, {ChIP-chip}, {PPI},
- {Time course, ChIP-chip}, {Time course, PPI}, {ChIP-chip, PPI}, and
- {Time course, ChIP-chip, PPI}.

The number of genes meeting this criteria between any two datasets is indicative of how strongly they influence each other and is expected to align with

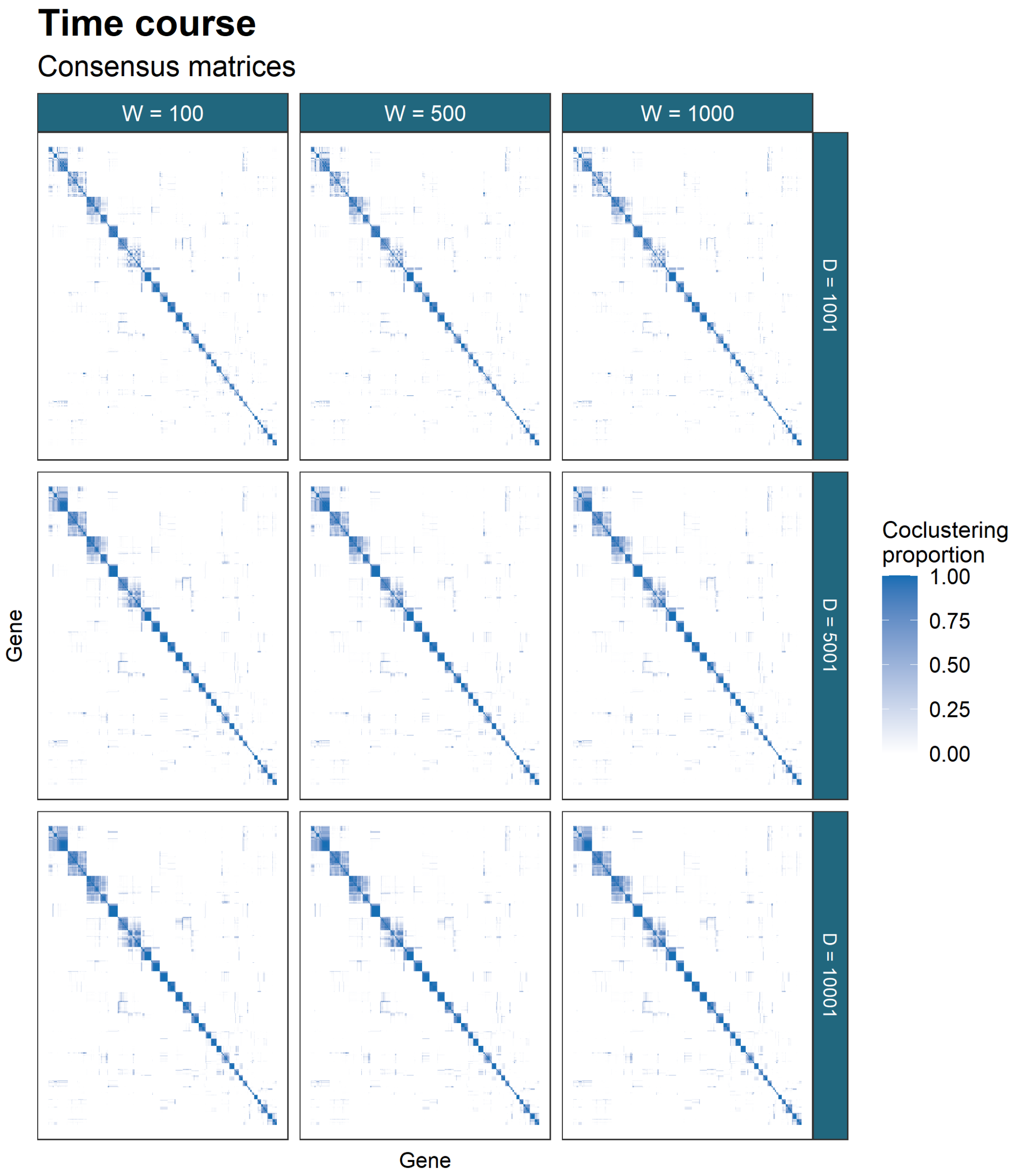

Figure S13: Consensus matrices for different ensembles of MDI for the time course data. This dataset has stable clustering across the different choices of number of chains, W, and chain depth, D, with some components merging as the chain depth increases.

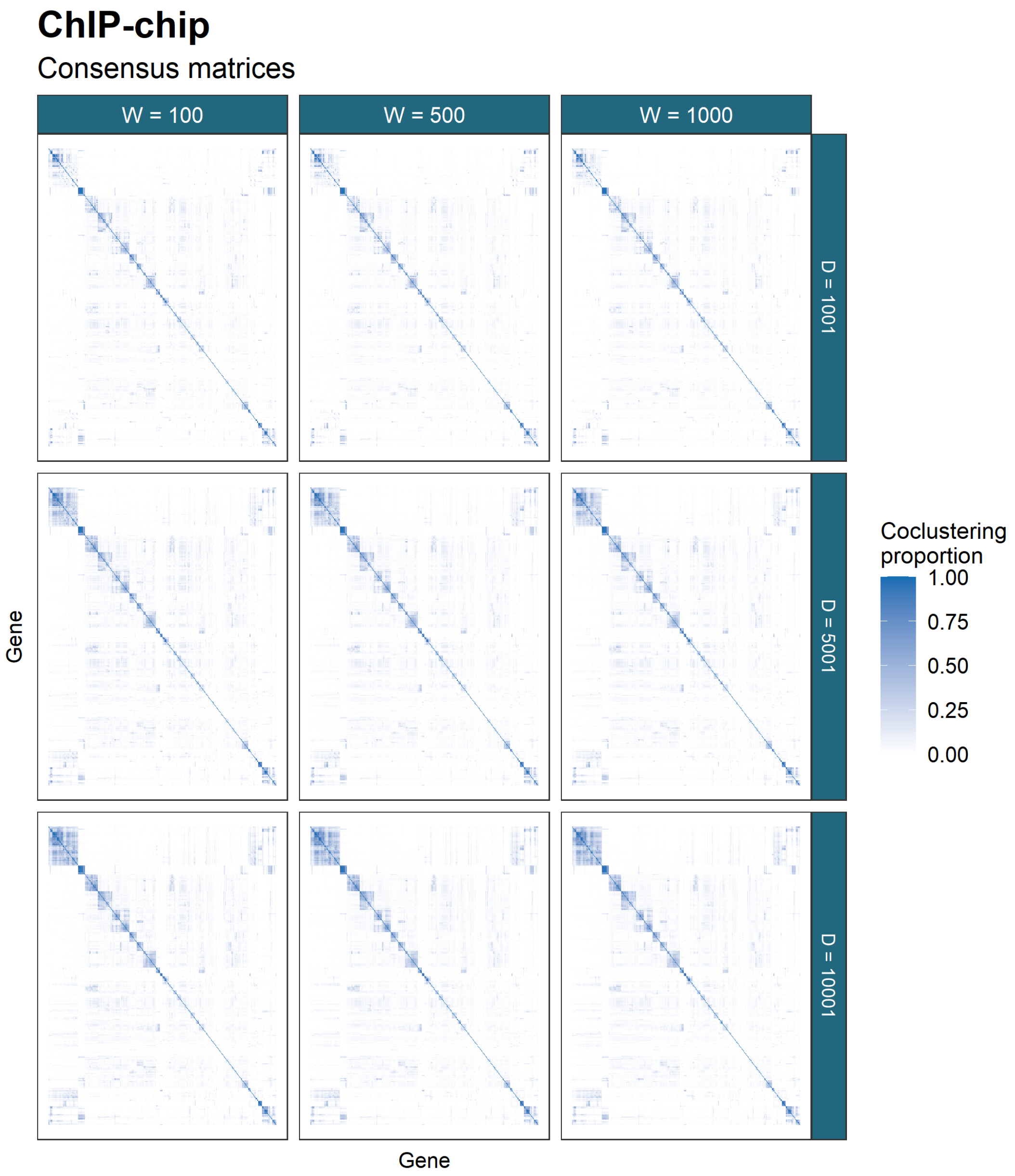

Figure S14: The ChIP-chip dataset is more sparse than the time course data. In keeping with the results from the simulations for mixture models, deeper chains are required for better performance. It is only between $D = 5,001$ and $D = 10,001$ that no change in the clustering can be observed and the result is believed to be stable. In this dataset the number of chains used, W, appears relatively unimportant, with similar results for $W = 100, 500, 1000$.

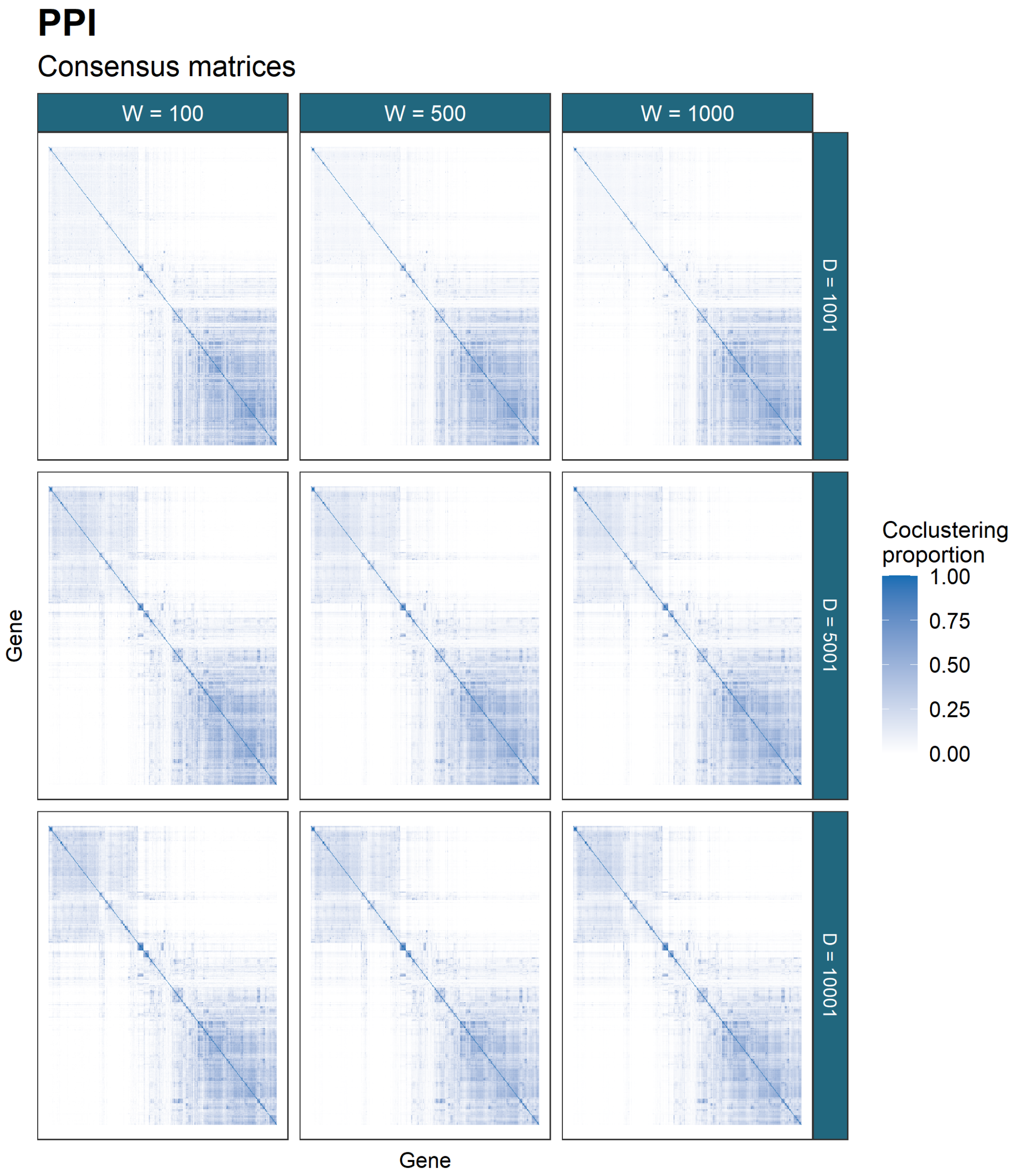

Figure S15: The PPI dataset has awkward characteristics for modelling. A wide, sparse dataset it is chain depth that we found to be the most important parameter for the ensemble. Similar to the results in figure S14, the matrices only stabilise from $D = 5001$ to $D = 10001$.

the ϕ_{lm} parameters from the MDI model. We find the following number of unique genes integrated between each combination of datasets:

- Time course + ChIP-chip + PPI: 56,
- Time course + ChIP-chip: 205 (261 including the 56 integrated across all datasets),
- Time course + PPI: 12 (68),
- ChIP-chip + PPI: 43 (99). .

This shows that the time course and ChIP-chip datasets contain very similar structure, the ChIP-chip and PPI datasets have some similarity but significantly less and the time course and PPI datasets have less shared signal again.

Compare this to the original analysis of this data in Kirk et al. (2012), where the number of such genes in each combination is:

- Time course + ChIP-chip + PPI: 16,
- Time course + ChIP-chip: 32 (48),
- Time course + PPI: 16 (32),
- ChIP-chip + PPI: 15 (31).

Our analysis has found significantly more shared structure.

5.1.1 Time course ChIP-chip analysis

We focus upon the dataset pairing of time course + ChIP-chip within the integrative analysis as the combination with the greatest number of genes with shared clustering. We show these genes grouped by their inferred cluster in figure S16. In this plot we exclude the 15 clusters where more than half of the member genes have no interactions in the ChIP-chip data and any clusters of one. We find that a small number of transcription factors dominate, with different combinations emerging across the 10 clusters shown here in table S2. Many of these 10 correspond to transcription factors that are well known to regulate cell cycle expression, namely MBP1, SWI4, SWI6, MCM1, FKH1, FKH2, NDD1, SWI5, and ACE2 (Simon et al., 2001).

Table S2: Table of transcription factors prominent in clusters of genes with shared labels for a majority of samples for the time course and ChIP-chip datasets.

Gene	Name	Description

YLR131C	ACE2	Transcription factor required for septum destruction after cytokinesis; phosphorylation by Cbk1p blocks nuclear exit during M/G1 transition; phosphorylation by cyclins Cdc28p and Pho85p prevents nuclear import during cell cycle phases other than cytokinesis; part of RAM network that regulates cellular polarity and morphogenesis; ACE2 has a paralog, SWI5, that arose from the whole genome duplication
YPL049C	DIG1	MAP kinase-responsive inhibitor of the Ste12p transcription factor; involved in the regulation of mating-specific genes and the invasive growth pathway; Dig1p and paralog Dig2p bind to Ste12p
YIL131C	FKH1	Forkhead family transcription factor; evolutionarily conserved lifespan regulator; binds multiple chromosomal elements with distinct specificities, cell cycle dynamics; regulates transcription elongation, chromatin silencing at mating loci, expression of G2/M phase genes
YNL068C	FKH2	Forkhead family transcription factor; rate-limiting activator of replication origins; evolutionarily conserved regulator of lifespan; binds multiple chromosomal elements with distinct specificities, cell cycle dynamics; positively regulates transcriptional elongation; negative role in chromatin silencing at HML and HMR; major role in expression of G2/M phase genes
YDL056W	MBP1	Transcription factor; involved in regulation of cell cycle progression from G1 to S phase, forms a complex with Swi6p that binds to MluI cell cycle box regulatory element in promoters of DNA synthesis genes
YMR043W	MCM1	Transcription factor; involved in cell-type-specific transcription and pheromone response; plays a central role in the formation of both repressor and activator complexes; involved in the transcription of some M/G1 genes Simon et al. (2001).
YOR372C	NDD1	Transcriptional activator essential for nuclear division; essential component of the mechanism that activates the expression of a set of late-S-phase-specific genes; turnover is tightly regulated during cell cycle and in response to DNA damage
YHR084W	STE12	Transcription factor that is activated by a MAPK signaling cascade; activates genes involved in mating or pseudohyphal/invasive growth pathways; cooperates with Tec1p transcription factor to regulate genes specific for invasive growth

YER111C	SWI4	DNA binding component of the SBF complex (Swi4p-Swi6p); a transcriptional activator that in concert with MBF (Mbp1-Swi6p) regulates late G1-specific transcription of targets including cyclins and genes required for DNA synthesis and repair
YDR146C	SWI5	Transcription factor that recruits Mediator and Swi/Snf complexes; activates transcription of genes expressed at the M/G1 phase boundary and in G1 phase; required for expression of the HO gene controlling mating type switching; localization to nucleus occurs during G1 and appears to be regulated by phosphorylation by Cdc28p kinase; SWI5 has a paralog, ACE2, that arose from the whole genome duplication
YLR182W	SWI6	Transcription cofactor; forms complexes with Swi4p (SBF) and Mbp1p (MBF) to regulate transcription at the G1/S transition (Simon et al., 2001); involved in meiotic gene expression; also binds Stb1p to regulate transcription at START; also required for the unfolded protein response, independently of its known transcriptional coactivators
YBR083W	TEC1	Transcription factor targeting filamentation genes and Ty1 expression; Ste12p activation of most filamentation gene promoters depends on Tec1p and Tec1p transcriptional activity is dependent on its association with Ste12p; binds to TCS elements upstream of filamentation genes, which are regulated by Tec1p/Ste12p/Dig1p complex; competes with Dig2p for binding to Ste12p/Dig1p; positive regulator of chronological life span
YML027W	YOX1	Homeobox transcriptional repressor; binds to Mcm1p and to early cell cycle boxes (ECBs) in the promoters of cell cycle-regulated genes expressed in M/G1 phase; phosphorylated by the cyclin Cdc28p; relocalizes from nucleus to cytoplasm upon DNA replication stress

These regulatory proteins are found in different combinations across the clusters. Based upon these combinations we associate each cluster with phases of the cell cycle and or some specific processes.

- Cluster 1: both ACE2 and SWI5 emerge. These regulate specific genes at the end of M and early G1 (McBride et al., 1999; Simon et al., 2001).
- Cluster 2: SWI5. This is similar to cluster 1, as ACE2 is a paralog of SWI5; therefore associated with M/G1. Furthermore, inspection of the expression in the timecourse data shows that the members of cluster 2

largely differentiate from those of cluster 1 based upon amplitude, not periodicity, suggesting that these clusters could be merged.

- Cluster 5: MBP1, SWI4 and SWI6. The SBF complex (Swi4p-Swi6p) is a transcriptional activator that in concert with MBF (Mbp1-Swi6p) regulates late G1-specific transcription of targets including cyclins and genes required for DNA synthesis and repair, controlling the transition to S phase (Simon et al., 2001; Iyer et al., 2001; Aligianni et al., 2009).
- Cluster 9: MBP1 and SWI6. These combine to form MBF, which regulates DNA replication and repair (Iyer et al., 2001).
- Cluster 11: DIG1, SWI4, SWI6, and STE12 emerge in all members with some having associations with TEC1. TEC1 and STE12, controls development, including cell adhesion and filament formation and is negatively regulated by DIG1 and DIG2 (van der Felden et al., 2014).
- Cluster 12: MBP1 , SWI4 and SWI6. Similar to cluster 5 in both the time course and ChIP-chip datasets and thus G1/S phase.
- Cluster 16: some MBP1, SWI4 and SWI6. The constituents of this cluster are largely associated with proteins contributing to histones H1, H2A, H2B, H3 and H4, suggesting an S-phase cluster (Ewen, 2000).
- Cluster 17: FKH1 and FKH2. Fkh1p and Fkh2p are required for cell-cycle regulation of transcription during G2/M (Kumar et al., 2000).
- Cluster 20: NDD1 and MCM1 with some FKH2. Mcm1, together with Fkh1 or Fkh2, recruits the Ndd1 protein in late G2, and thus controls the transcription of G2/M genes (Simon et al., 2001; Koranda et al., 2000).
- Cluster 26: YOX1 and MCM1. YOX1 binds to Mcm1p and to early cell cycle boxes (ECBs) in the promoters of cell cycle-regulated genes expressed in M/G1 phase (Pramila et al., 2002).

Gene	Name	Cluster	Description
YJL115W	ASF1	9	Nucleosome assembly factor; involved in chromatin assembly, disassembly; required for buffering mRNA synthesis rate against gene dosage changes in S phase
YLR103C	CDC45	9	DNA replication initiation factor; recruited to MCM pre-RC complexes at replication origins; recruits elongation machinery; binds tightly to ssDNA, which disrupts interaction with the MCM helicase and stalls it during replication stress; mutants in human homolog may cause velocardiofacial and DiGeorge syndromes

YPL241C	CIN2	9	GTPase-activating protein (GAP) for Cin4p; tubulin folding factor C involved in beta-tubulin (Tub2p) folding; mutants display increased chromosome loss and benomyl sensitivity; human homolog RP2 complements yeast null mutant
YPR175W	DPB2	9	Second largest subunit of DNA polymerase II (DNA polymerase epsilon); required for maintenance of fidelity of chromosomal replication; essential motif in C-terminus is required for formation of the four-subunit Pol epsilon; expression peaks at the G1/S phase boundary; Cdc28p substrate
YIL026C	IRR1	9	Subunit of the cohesin complex; which is required for sister chromatid cohesion during mitosis and meiosis and interacts with centromeres and chromosome arms
YCL061C	MRC1	9	S-phase checkpoint protein required for DNA replication; couples DNA helicase and polymerase; defines a novel S-phase checkpoint with Hog1p that coordinates DNA replication and transcription upon osmostress; protects uncapped telomeres; Dia2p-dependent degradation mediates checkpoint recovery; mammalian claspin homolog; subunit of a replication-pausing checkpoint complex, Tof1p-Mrc1p-Csm3p; checkpoint-mediator protein that functions during DNA replication and activates the effector kinase Rad53 (Bando et al., 2009); human ATR homolog (Lao et al., 2018)
YDR097C	MSH6	9	Protein required for mismatch repair in mitosis and meiosis; forms a complex with Msh2p to repair both single-base and insertion-deletion mispairs; also involved in interstrand cross-link repair; potentially phosphorylated by Cdc28p
YNL102W	POL1	9	Catalytic subunit of the DNA polymerase I alpha-primase complex; required for the initiation of DNA replication during mitotic DNA synthesis and premeiotic DNA synthesis
YBL035C	POL12	9	B subunit of DNA polymerase alpha-primase complex; required for initiation of DNA replication during mitotic and premeiotic DNA synthesis; also functions in telomere capping and length regulation
YKL113C	RAD27	9	5' to 3' exonuclease, 5' flap endonuclease; required for Okazaki fragment processing and maturation, for long-patch base-excision repair and large loop repair (LLR), ribonucleotide excision repair
YPL153C	RAD53	9	DNA damage response protein kinase; required for cell-cycle arrest, regulation of copper genes in response to DNA damage; human homolog CHEK2 implicated in breast cancer can complement yeast null mutant

YAR007C	RFA1	9	Subunit of heterotrimeric Replication Protein A (RPA); RPA is a highly conserved single-stranded DNA binding protein involved in DNA replication, repair, and recombination; RPA protects against inappropriate telomere recombination, and upon telomere uncapping, prevents cell proliferation by a checkpoint-independent pathway; role in DNA catenation/decatenation pathway of chromosome disentangling; relocalizes to the cytosol in response to hypoxia
YNL312W	RFA2	9	Subunit of heterotrimeric Replication Protein A (RPA); RPA is a highly conserved single-stranded DNA binding protein involved in DNA replication, repair, and recombination; RPA protects against inappropriate telomere recombination, and upon telomere uncapping, prevents cell proliferation by a checkpoint-independent pathway; in concert with Sgs1p-Top2p-Rmi1p, stimulates DNA catenation/decatenation activity of Top3p; protein abundance increases in response to DNA replication s
YAR008W	SEN34	9	Subunit of the tRNA splicing endonuclease; tRNA splicing endonuclease (Sen complex) is composed of Sen2p, Sen15p, Sen34p, and Sen54p; Sen complex also cleaves the CBP1 mRNA at the mitochondrial surface; Sen34p contains the active site for tRNA 3' splice site cleavage and has similarity to Sen2p and to Archaeal tRNA splicing endonuclease
YJL074C	SMC3	9	Subunit of the multiprotein cohesin complex; required for sister chromatid cohesion in mitotic cells; also required, with Rec8p, for cohesion and recombination during meiosis; phylogenetically conserved SMC chromosomal ATPase family member
YNL273W	TOF1	9	Subunit of a replication-pausing checkpoint complex; Tof1p-Mrc1p-Csm3p acts at the stalled replication fork to promote sister chromatid cohesion after DNA damage, facilitating gap repair of damaged DNA; interacts with the MCM helicase; checkpoint-mediator protein that functions during DNA replication and activates the effector kinase RAD53 (Bando et al., 2009); human ATM homolog (Lao et al., 2018)
YMR215W	GAS3	16	Putative 1,3-beta-glucanosyltransferase; has similarity go other GAS family members; low abundance, possibly inactive member of the GAS family of GPI-containing proteins; localizes to the cell wall; mRNA induced during sporulation

YBR009C	HHF1	16	Histone H4; core histone protein required for chromatin assembly and chromosome function; one of two identical histone proteins (see also HHF2); contributes to telomeric silencing; N-terminal domain involved in maintaining genomic integrity
YNL030W	HHF2	16	Histone H4; core histone protein required for chromatin assembly and chromosome function; one of two identical histone proteins (see also HHF1); contributes to telomeric silencing; N-terminal domain involved in maintaining genomic integrity
YPL127C	HHO1	16	Histone H1, linker histone with roles in meiosis and sporulation; decreasing levels early in sporulation may promote meiosis, and increasing levels during sporulation facilitate compaction of spore chromatin; binds to promoters and within genes in mature spores; may be recruited by Ume6p to promoter regions, contributing to transcriptional repression outside of meiosis; suppresses DNA repair involving homologous recombination
YBR010W	HHT1	16	Histone H3; core histone protein required for chromatin assembly, part of heterochromatin-mediated telomeric and HM silencing; one of two identical histone H3 proteins (see HHT2); regulated by acetylation, methylation, and phosphorylation; H3K14 acetylation plays an important role in the unfolding of strongly positioned nucleosomes during repair of UV damage
YNL031C	HHT2	16	Histone H3; core histone protein required for chromatin assembly, part of heterochromatin-mediated telomeric and HM silencing; one of two identical histone H3 proteins (see HHT1); regulated by acetylation, methylation, and phosphorylation; H3K14 acetylation plays an important role in the unfolding of strongly positioned nucleosomes during repair of UV damage
YDR225W	HTA1	16	Histone H2A; core histone protein required for chromatin assembly and chromosome function; one of two nearly identical subtypes (see also HTA2); DNA damage-dependent phosphorylation by Mec1p facilitates DNA repair; acetylated by Nat4p; N-terminally propionylated in vivo
YBL003C	HTA2	16	Histone H2A; core histone protein required for chromatin assembly and chromosome function; one of two nearly identical (see also HTA1) subtypes; DNA damage-dependent phosphorylation by Mec1p facilitates DNA repair; acetylated by Nat4p

YDR224C	HTB1	16	Histone H2B; core histone protein required for chromatin assembly and chromosome function; nearly identical to HTB2; Rad6p-Bre1p-Lge1p mediated ubiquitination regulates reassembly after DNA replication, transcriptional activation, meiotic DSB formation and H3 methylation
YBL002W	HTB2	16	Histone H2B; core histone protein required for chromatin assembly and chromosome function; nearly identical to HTB1; Rad6p-Bre1p-Lge1p mediated ubiquitination regulates reassembly after DNA replication, transcriptional activation, meiotic DSB formation and H3 methylation
YNR009W	NRM1	16	Transcriptional co-repressor of MBF-regulated gene expression; Nrm1p associates stably with promoters via MCB binding factor (MBF) to repress transcription upon exit from G1 phase
YDR113C	PDS1	16	Securin; inhibits anaphase by binding separin Esp1p; blocks cyclin destruction and mitotic exit, essential for meiotic progression and mitotic cell cycle arrest; localization is cell-cycle dependent and regulated by Cdc28p phosphorylation

Table S3: Description of the genes with common labelling across the time course and ChIP-chip datasets from clusters 9 and 16.

5.2 Bayesian analysis

We wished to compare our results from consensus clustering to a conventional Bayesian approach. We ran 10 chains of MDI for 36 hours saving every thousandth sample. This resulted in chains of varying length. We reduced the chains to 666 samples as this was the number of samples achieved by the shortest chain. Similar to section 4.3 these chains were then investigated for

- within-chain stationarity using the Geweke convergence diagnostic (Geweke et al., 1991), and
- across-chain convergence using $\hat{R}$ (Gelman et al., 1992) and the Vats-Knudson extension (*stable* $\hat{R}$, Vats and Knudson, 2018).

Again we focus upon stationarity of the continuous variables. In the implementation of MDI we used, the recorded continuous variables are the concentration parameters of the Dirichlet distribution for the dataset-specific component weights and the ϕ_{ij} parameter associated with the correlation between the ith and j^{th} datasets.

We plot the Geweke-statistic for each chain in figure S17. No chain is perfectly behaved; as we cannot reduce to the set of stationary chains we thus exclude the most poorly behaved chains. Our lack of belief in the convergence

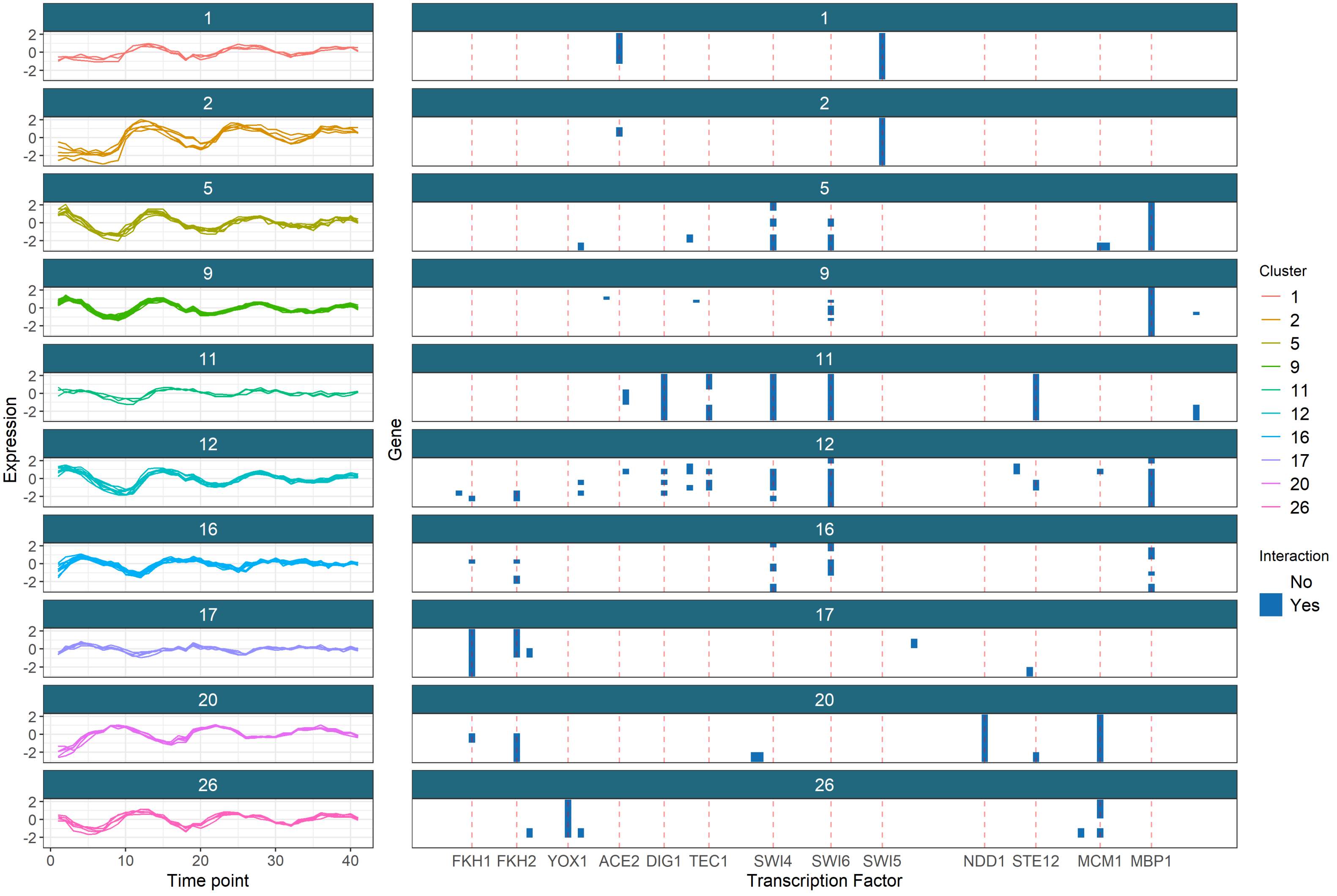

Figure S16: The clusters of genes with common labels across the time course and ChIP-chip datasets (as described in tbale ??). We exclude the clusters with no interactions in the ChIP-chip dataset and include a red line for the Transcription factors that dominate the clustering structure in the ChIP-chip dataset.

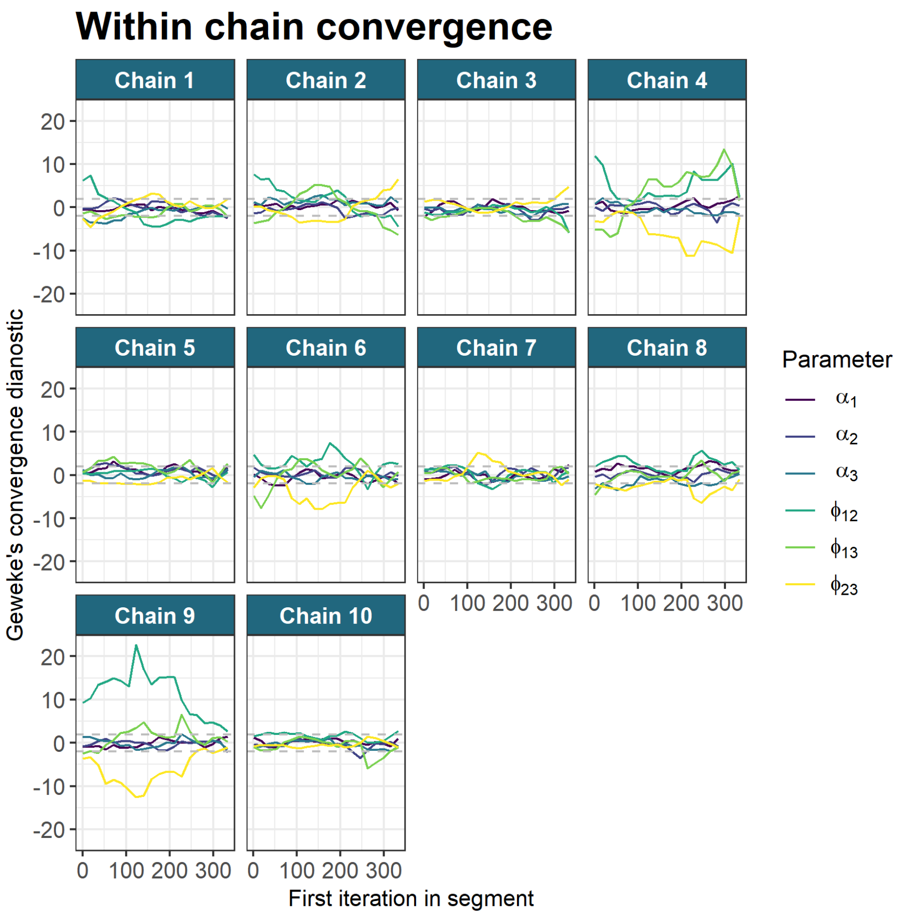

Figure S17: Chain 9 can be seen to have the most extreme behaviour in the distribution of the Geweke diagnostic for the parameters. We remove this chain from the analysis. Of the remaining chains we believe that 1, 2, 4 and 6 express the distributions furthest removed from the desired behaviour and are dropped from the analysis.

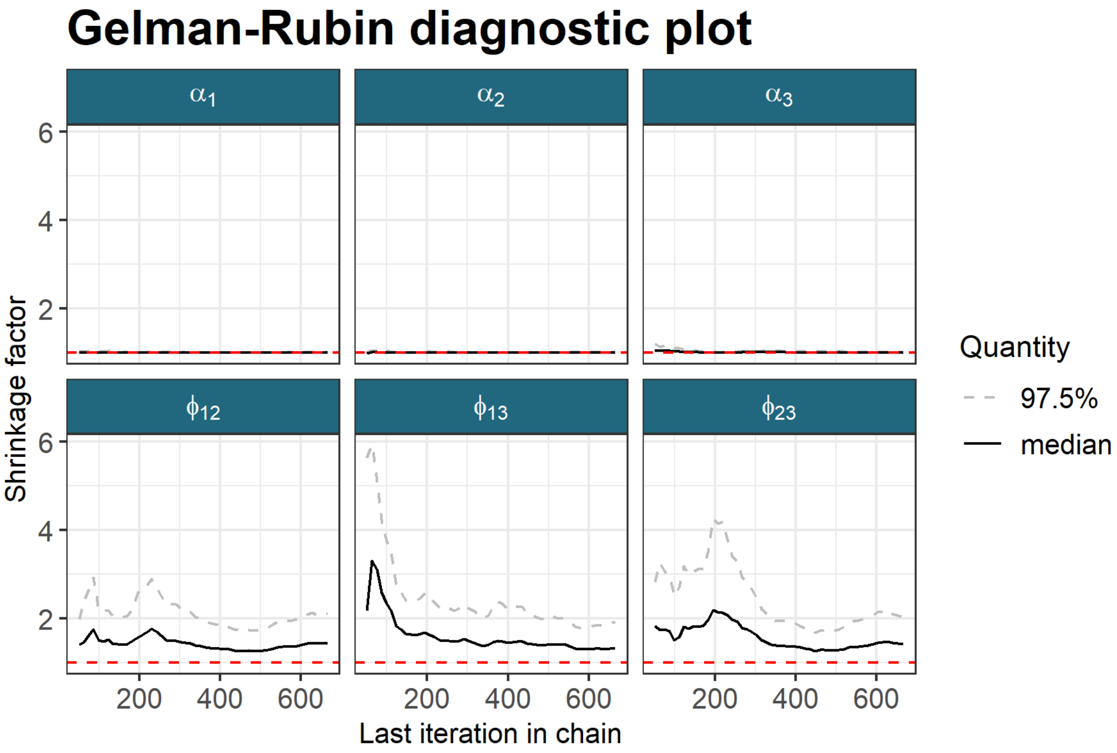

Figure S18: The chains still appear to be unconverged with $\hat{R}$ remaining above 1.25 for the ϕ_{12}, ϕ_{13} and ϕ_{23} parameters. Stable $\hat{R}$ is also too high with values of 1.049, 1.052 and 1.057 for ϕ_{12}, ϕ_{13} and ϕ_{23} respectively. The values of α_l cannot be seen due to the scaling of the y-axis.

of these chains is fortified by the behaviour of $\hat{R}$ (which can be seen in figure S18) and the different distributions sampled for the ϕ_{lm} parameters shown in figure S19.

We visualise the the PSMs for each dataset in figure S20.

If we compare the distribution of sampled values for the ϕ parameters for the Bayesian chains that we keep based upon their convergence diagnostics, the final ensemble used ($D = 10001$, $W = 1000$) and the pooled samples from the 5 long chains, then we see that the ensemble consisting of the long chains (which might be believed to sampling different parts of the posterior distribution) is closer in its appearance to the distributions sampled by the consensus clustering than to any single chain.

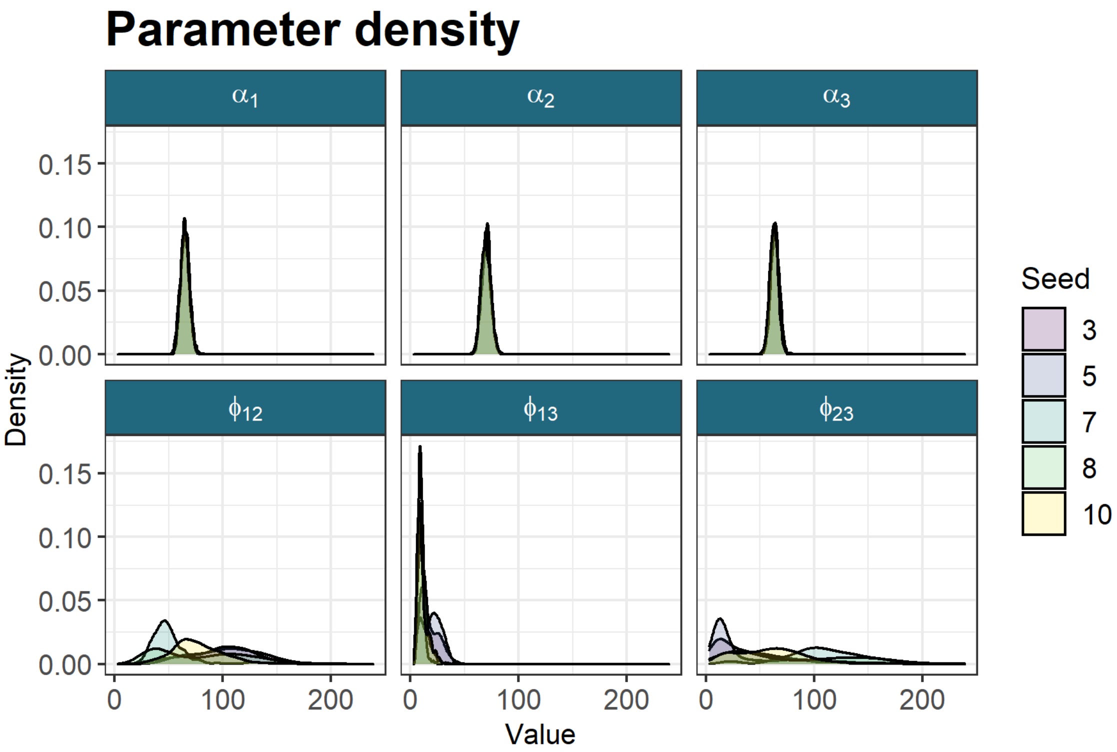

Figure S19: The densities of the continuous variables across the 5 chains kept for analysis. The mean sampled values are $\alpha_1 = 64.84$, $\alpha_2 = 69.85$, $\alpha_2 = 63.22$, $\phi_{12} = 81.76$, $\phi_{13} = 13.87$, and $\phi_{23} = 65.03$. It can be seen that different modes are being sampled for the ϕ parameters in each chain.

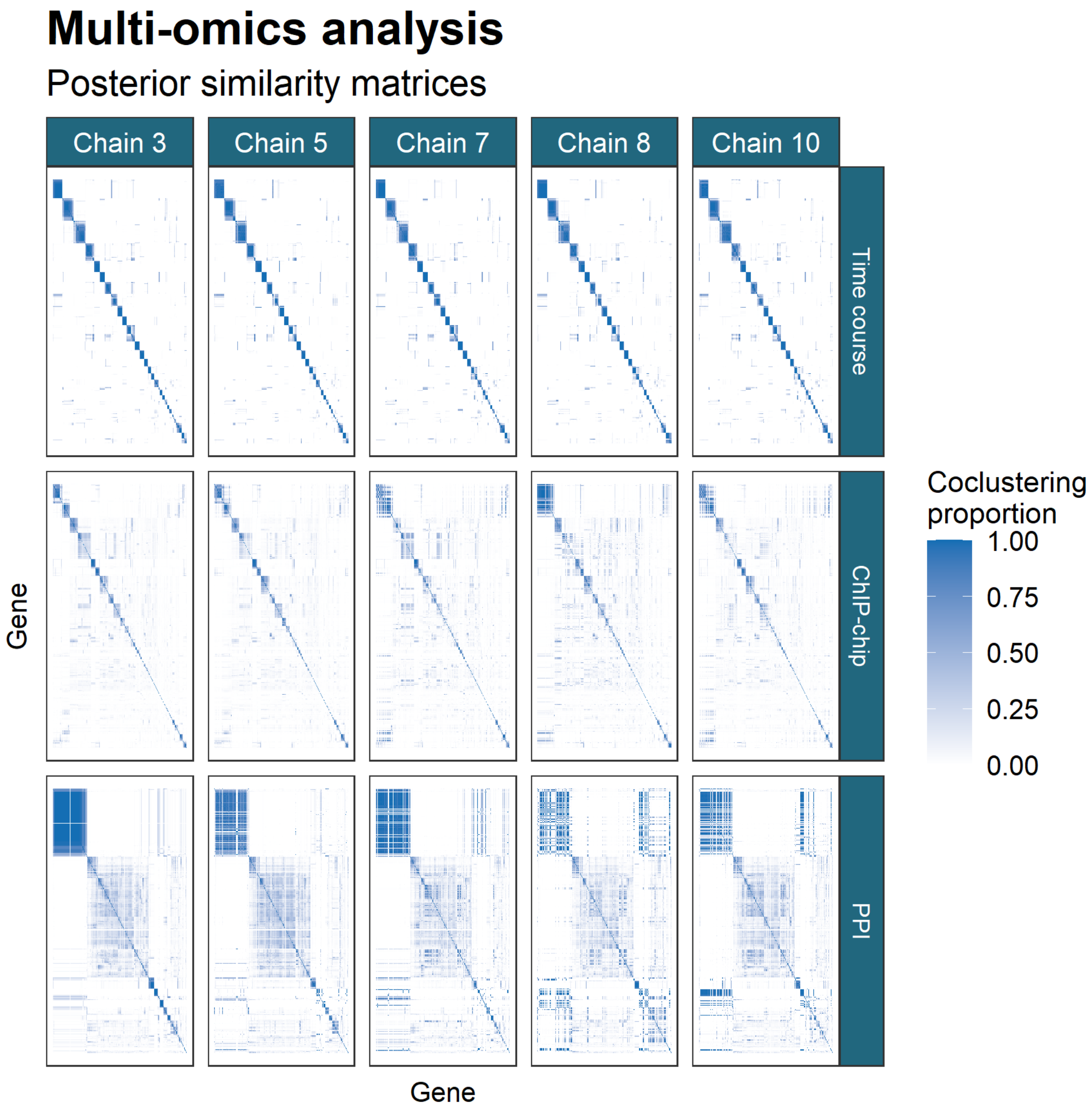

Figure S20: PSMs for each chain within each dataset. The PSMs are ordered by hierarchical clustering of the rows of the PSM for chain 3 in each dataset. There is no marked difference between the matrices for the time course data with disagreement becoming more prominent in the ChIP-chip data and more so again in the PPI dataset.

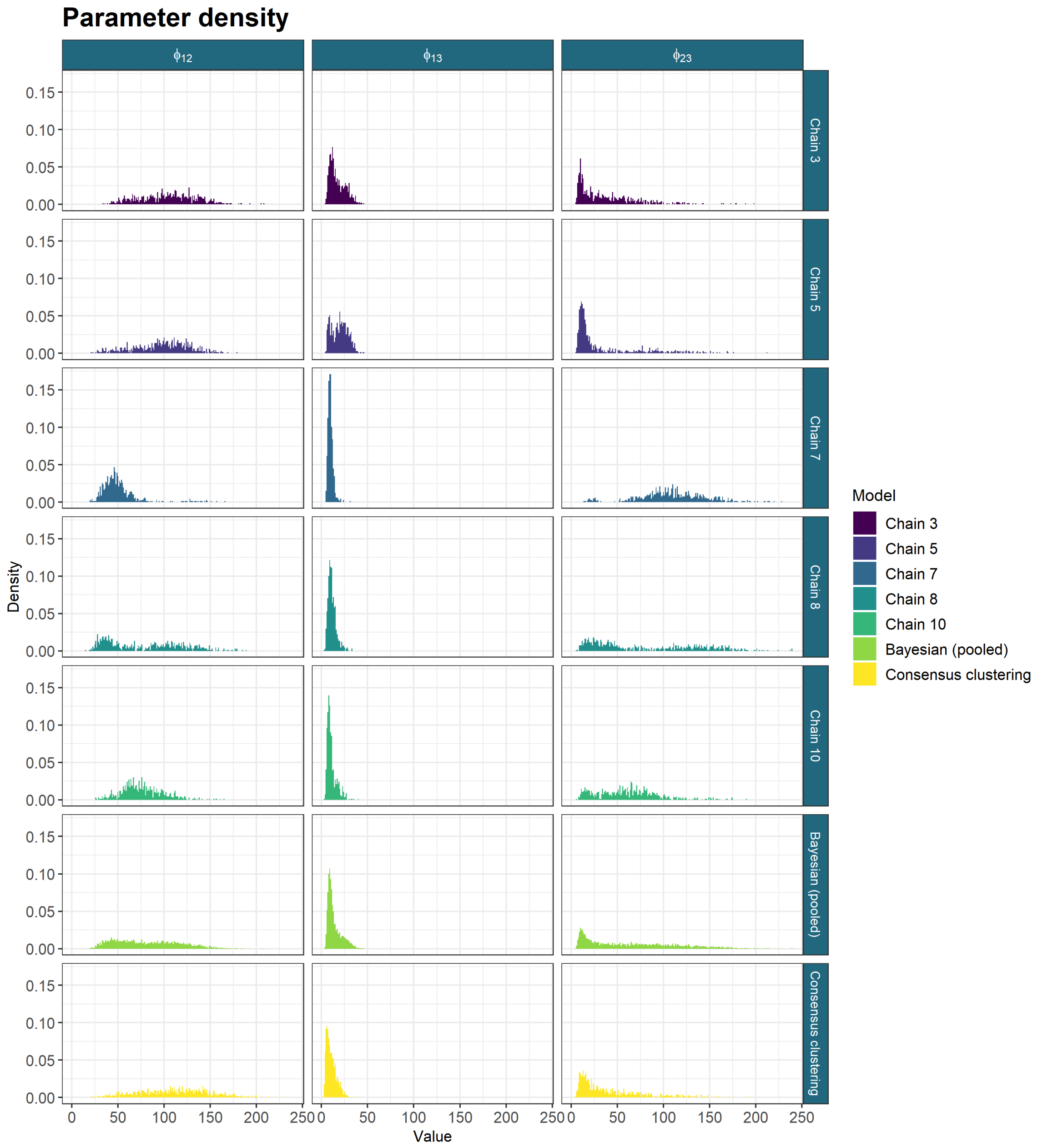

Figure S21: The sampled values for the ϕ parameters from the long chains, their pooled samples and the consensus using 1000 chains of depth 10,001. The long chains display a variety of behaviours. Across chains there is no clear consensus on the nature of the posterior distribution. The samples from any single chain are not particularly close to the behaviour of the pooled samples across all three parameters. It is the consensus clustering that most approaches this pooled behaviour.

5.3 GO term over-representation

We further show the lack of disagreement between the long chains from section 5.2 in a Gene Ontology (GO) term over-representation analysis. We estimated clusterings from the PSMs of the chains kept from section 5.2 visualised in figure S20 and the consensus matrix of the largest ensemble run (i.e. $CC(10001, 1000)$) using the `maxpear` function from the R package `mcclust` Fritsch (2012) using default settings except for `k.max` which was set to $275 \approx N/2$. To perform the GO term over-representation analysis we used the `Bioconductor` packages `clusterProfiler` (Yu et al., 2012), `biomaRt` (Durinck et al., 2009) and the annotation package `org.Sc.sgd.db` (Carlson et al., 2014).

We conditioned the test on the background set of the 551 yeast genes in the data. The gene labelled YIL167W was not found in the annotation database and was dropped from the analysis leaving a background universe of 550 genes. A hypergeometric test was used to check if the number of genes associated with specific GO terms within a cluster was greater than expected by random chance. We corrected the p-values using the Benjamini-Hochberg correction (Benjamini and Hochberg, 1995) and defined significance by a threshold of 0.01. We plotted the over-represented GO terms for the different clusterings within each dataset using the three different ontologies of "Molecular function" (**MF**), "Biological process" (**BP**) and "Cellular component" (**CC**) (figures S22, S23 and S24 respectively).

As we expect based upon the disagreement shown in figure S21, we find that the Bayesian chains have very significant disagreements between each other; there is no consensus on the results with many terms enriched in one or two chains. However, the consensus clustering finds many of the terms common to all of the long chains. This is what we would expect based upon the similarity of the ϕ_{lm} distribution in the ensemble and the pooled long chains. Consensus clustering also finds some terms with low p-values common to a majority of chains (such as DNA helicase activity in the MF ontology for the time course dataset) and a small number of GO terms unique to itself. These terms that no long chain find are normally related to other terms already over-represented within either the consensus clustering or a number of the long chains. For example, the transmembrane transporter activity and transporter activity terms uncovered by the ensemble in the time course dataset are related to terms found across 3 of the chains and by consensus clustering (specifically transferase activity and phosphotransferase).

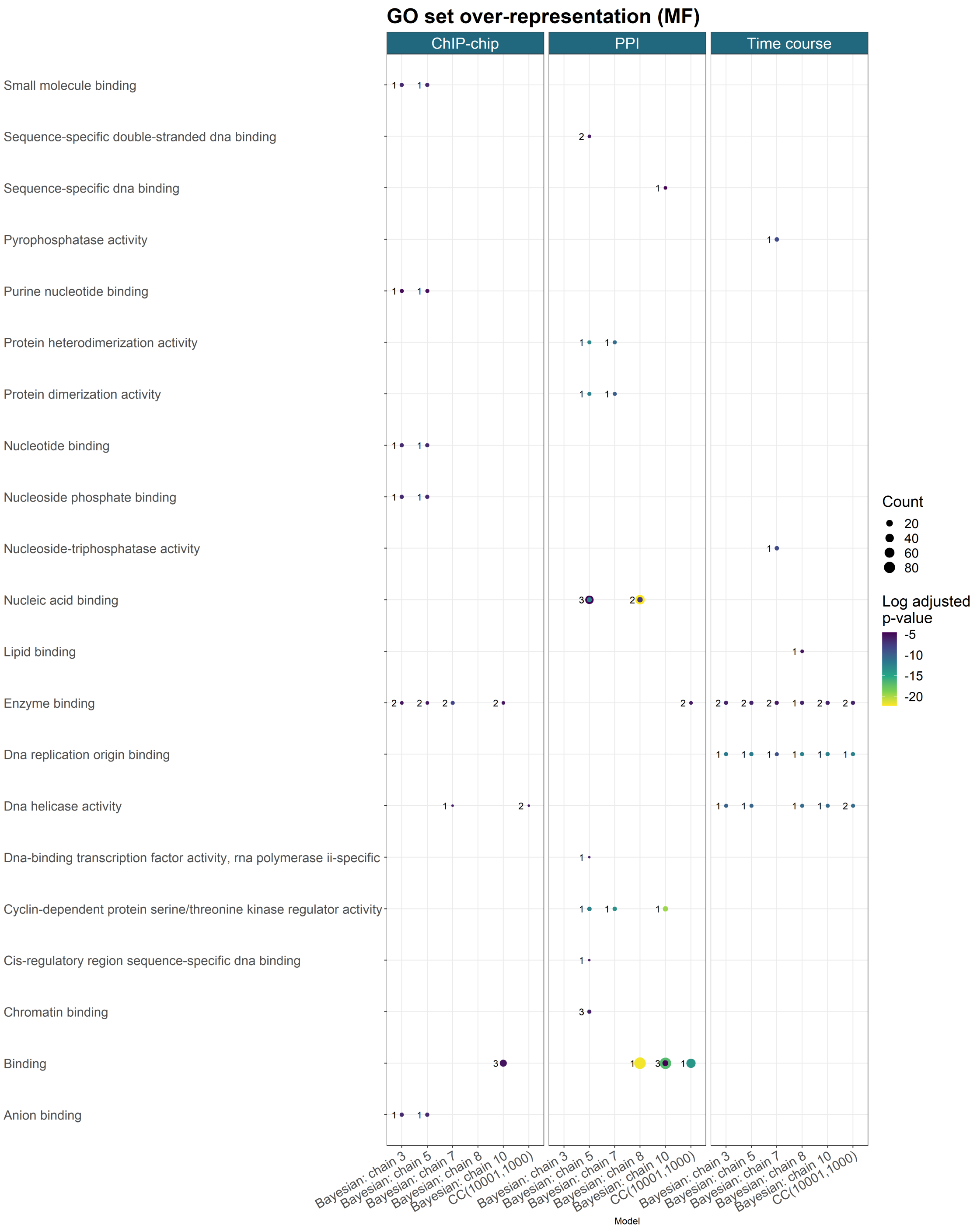

Figure S22: GO term over-representation for the Molecular function ontology for each dataset from the final clustering of each method.

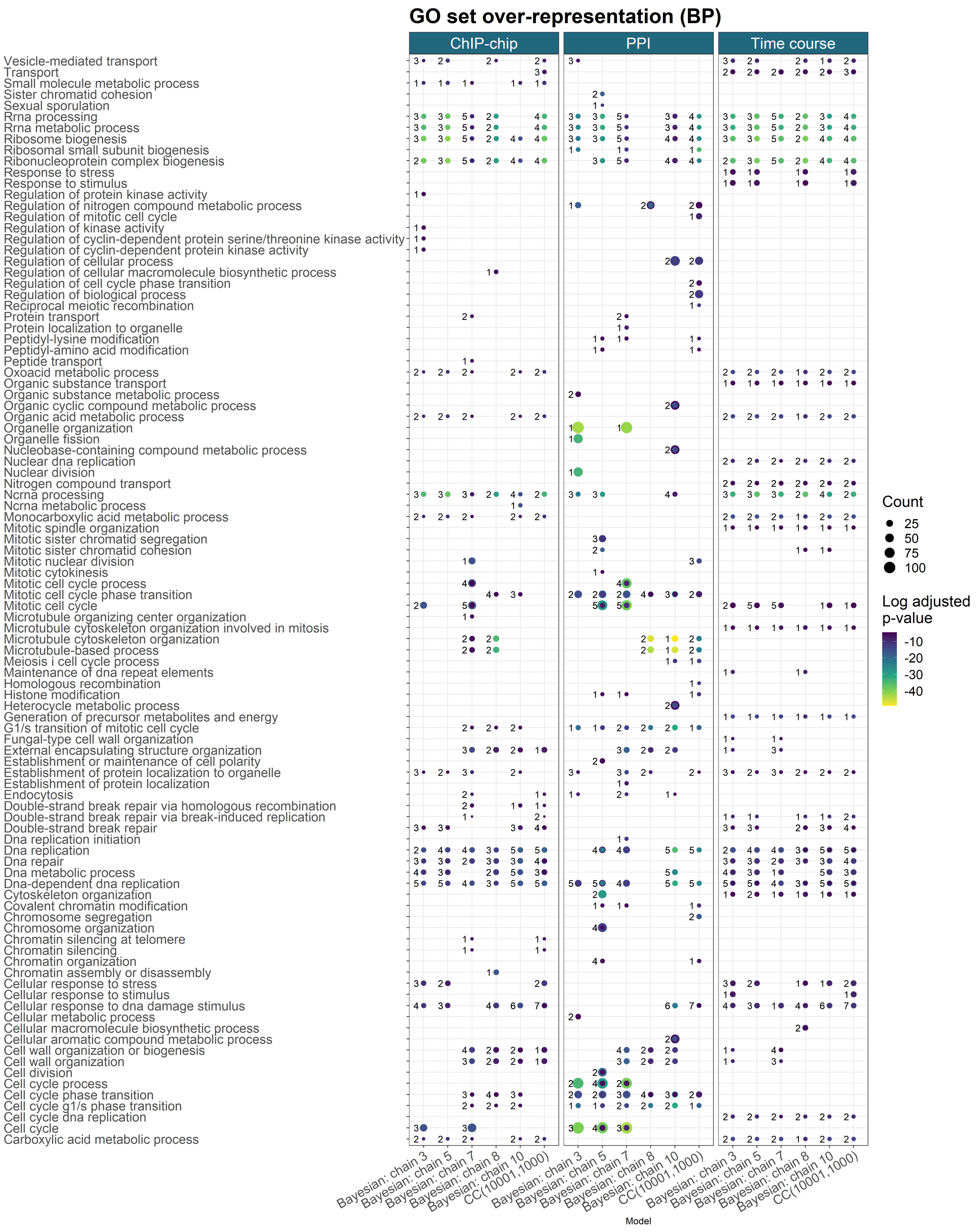

Figure S23: GO term over-representation for the Biological process ontology for each dataset from the final clustering of each method.

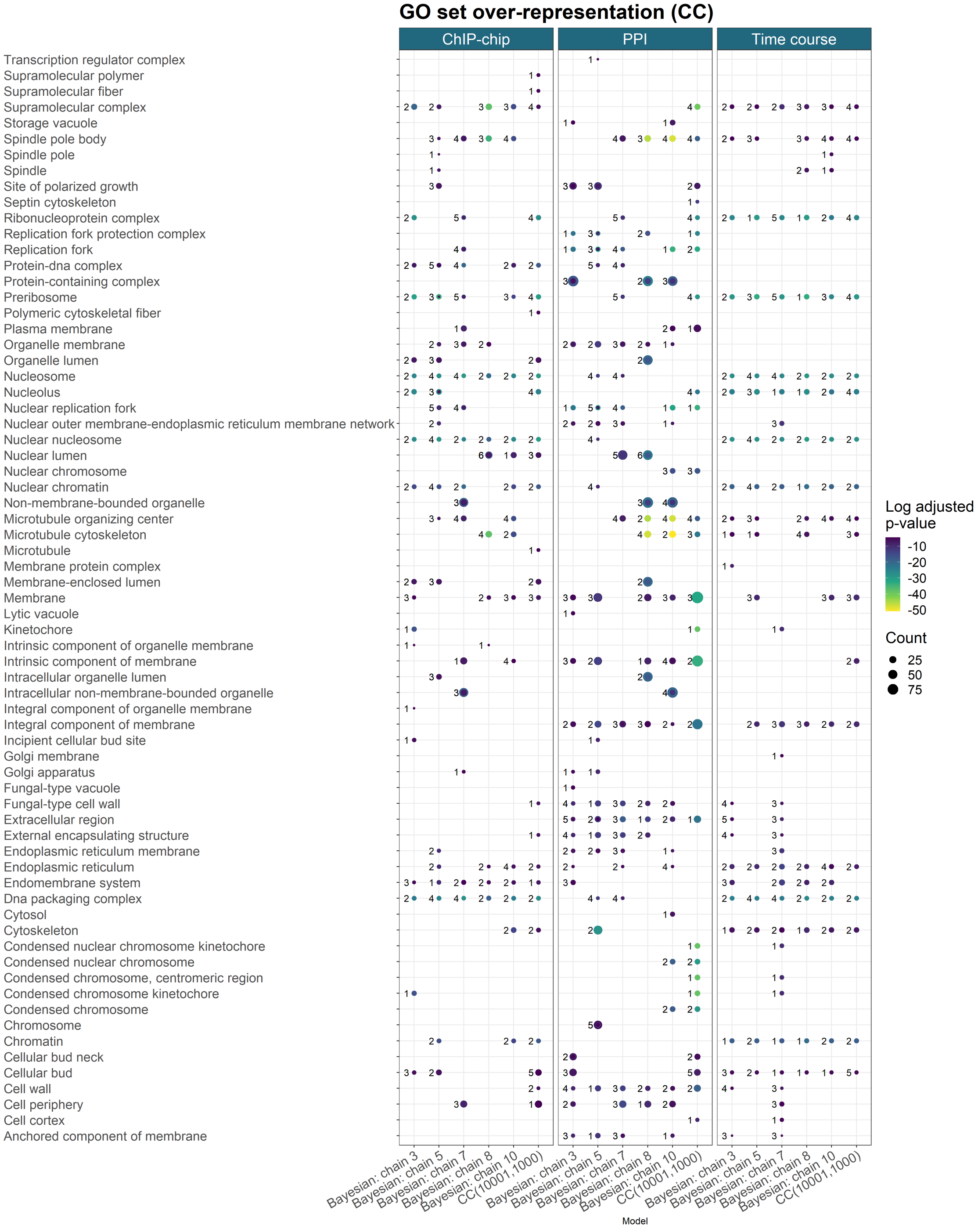

Figure S24: GO term over-representation for the Cellular component ontology for each dataset from the final clustering of each method.

References

Sofia Aligianni, Daniel H Lackner, Steffi Klier, Gabriella Rustici, Brian T Wilhelm, Samuel Marguerat, Sandra Codlin, Alvis Brazma, Robertus AM de Bruin, and Jürg Bähler. The fission yeast homeodomain protein yox1p binds to mbf and confines mbf-dependent cell-cycle transcription to g1-s via negative feedback. *PLoS Genet*, 5(8):e1000626, 2009.

Masashige Bando, Yuki Katou, Makiko Komata, Hirokazu Tanaka, Takehiko Itoh, Takashi Sutani, and Katsuhiko Shirahige. Csm3, tof1, and mrc1 form a heterotrimeric mediator complex that associates with dna replication forks. *Journal of Biological Chemistry*, 284(49):34355–34365, 2009.

Yoav Benjamini and Yosef Hochberg. Controlling the false discovery rate: a practical and powerful approach to multiple testing. *Journal of the Royal statistical society: series B (Methodological)*, 57(1):289–300, 1995.

M Carlson, S Falcon, H Pages, and N Li. Org. sc. sgd. db: Genome wide annotation for yeast. *R package version*, 2(1), 2014.

Raymond J Cho, Michael J Campbell, Elizabeth A Winzeler, Lars Steinmetz, Andrew Conway, Lisa Wodicka, Tyra G Wolfsberg, Andrei E Gabrielian, David Landsman, David J Lockhart, et al. A genome-wide transcriptional analysis of the mitotic cell cycle. *Molecular cell*, 2(1):65–73, 1998.

Steffen Durinck, Paul T Spellman, Ewan Birney, and Wolfgang Huber. Mapping identifiers for the integration of genomic datasets with the r/bioconductor package biomart. *Nature protocols*, 4(8):1184, 2009.

Mark E Ewen. Where the cell cycle and histones meet. *Genes & development*, 14(18):2265–2270, 2000.

Thomas S Ferguson. A bayesian analysis of some nonparametric problems. *The annals of statistics*, pages 209–230, 1973.

Arno Fritsch. *mcclust: Process an MCMC Sample of Clusterings*, 2012. URL `https://CRAN.R-project.org/package=mcclust`. R package version 1.0.

Arno Fritsch, Katja Ickstadt, et al. Improved criteria for clustering based on the posterior similarity matrix. *Bayesian analysis*, 4(2):367–391, 2009.

Evelina Gabasova, John Reid, and Lorenz Wernisch. Clusternomics: Integrative context-dependent clustering for heterogeneous datasets. *PLoS computational biology*, 13(10):e1005781, 2017.

Andrew Gelman, Donald B Rubin, et al. Inference from iterative simulation using multiple sequences. *Statistical science*, 7(4):457–472, 1992.

Andrew Gelman, John B Carlin, Hal S Stern, David B Dunson, Aki Vehtari, and Donald B Rubin. *Bayesian data analysis.* CRC press, 2013.

John Geweke et al. *Evaluating the accuracy of sampling-based approaches to the calculation of posterior moments*, volume 196. Federal Reserve Bank of Minneapolis, Research Department Minneapolis, MN, 1991.

Vishwanath R Iyer, Christine E Horak, Charles S Scafe, David Botstein, Michael Snyder, and Patrick O Brown. Genomic binding sites of the yeast cell-cycle transcription factors sbf and mbf. *Nature*, 409(6819):533–538, 2001.

Paul Kirk, Jim E Griffin, Richard S Savage, Zoubin Ghahramani, and David L Wild. Bayesian correlated clustering to integrate multiple datasets. *Bioinformatics*, 28(24):3290–3297, 2012.

Christina Knudson and Dootika Vats. *stableGR: A Stable Gelman-Rubin Diagnostic for Markov Chain Monte Carlo*, 2020. URL `https://CRAN.R-project.org/package=stableGR`. R package version 1.0.

Manfred Koranda, Alexander Schleiffer, Lukas Endler, and Gustav Ammerer. Forkhead-like transcription factors recruit ndd1 to the chromatin of g2/m-specific promoters. *Nature*, 406(6791):94–98, 2000.

Raman Kumar, David M Reynolds, Andrej Shevchenko, Anna Shevchenko, Sherilyn D Goldstone, and Stephen Dalton. Forkhead transcription factors, fkh1p and fkh2p, collaborate with mcm1p to control transcription required for m-phase. *Current Biology*, 10(15):896–906, 2000.

Jessica P Lao, Katie M Ulrich, Jeffrey R Johnson, Billy W Newton, Ajay A Vashisht, James A Wohlschlegel, Nevan J Krogan, and David P Toczyski. The yeast dna damage checkpoint kinase rad53 targets the exoribonuclease, xrn1. *G3: Genes, Genomes, Genetics*, 8(12):3931–3944, 2018.

Samuel A Mason, Faiz Sayyid, Paul DW Kirk, Colin Starr, and David L Wild. Mdi-gpu: accelerating integrative modelling for genomic-scale data using gp-gpu computing. *Statistical Applications in Genetics and Molecular Biology*, 15(1):83–86, 2016.

Helen J McBride, Yaxin Yu, and David J Stillman. Distinct regions of the swi5 and ace2 transcription factors are required for specific gene activation. *Journal of Biological Chemistry*, 274(30):21029–21036, 1999.

Stefano Monti, Pablo Tamayo, Jill Mesirov, and Todd Golub. Consensus clustering: a resampling-based method for class discovery and visualization of gene expression microarray data. *Machine learning*, 52(1-2):91–118, 2003.

Tata Pramila, Shawna Miles, Debraj GuhaThakurta, Dave Jemiolo, and Linda L Breeden. Conserved homeodomain proteins interact with mads box protein mcm1 to restrict ecb-dependent transcription to the m/g1 phase of the cell cycle. *Genes & development*, 16(23):3034–3045, 2002.

Sylvia Richardson and Peter J Green. On bayesian analysis of mixtures with an unknown number of components. *Journal of the Royal Statistical Society: series B*, 59(4):731–792, 1997.

Judith Rousseau and Kerrie Mengersen. Asymptotic behaviour of the posterior distribution in overfitted mixture models. *Journal of the Royal Statistical Society: Series B (Statistical Methodology)*, 73(5):689–710, 2011.

Richard S Savage, Zoubin Ghahramani, Jim E Griffin, Bernard J De la Cruz, and David L Wild. Discovering transcriptional modules by bayesian data integration. *Bioinformatics*, 26(12):i158–i167, 2010.

Gideon Schwarz et al. Estimating the dimension of a model. *The annals of statistics*, 6(2):461–464, 1978.

Samuel Sanford Shapiro and Martin B Wilk. An analysis of variance test for normality (complete samples). *Biometrika*, 52(3/4):591–611, 1965.

Itamar Simon, John Barnett, Nancy Hannett, Christopher T Harbison, Nicola J Rinaldi, Thomas L Volkert, John J Wyrick, Julia Zeitlinger, David K Gifford, Tommi S Jaakkola, et al. Serial regulation of transcriptional regulators in the yeast cell cycle. *Cell*, 106(6):697–708, 2001.

Paul T Spellman, Gavin Sherlock, Michael Q Zhang, Vishwanath R Iyer, Kirk Anders, Michael B Eisen, Patrick O Brown, David Botstein, and Bruce Futcher. Comprehensive identification of cell cycle–regulated genes of the yeast saccharomyces cerevisiae by microarray hybridization. *Molecular biology of the cell*, 9(12):3273–3297, 1998.

Julia van der Felden, Sarah Weisser, Stefan Brückner, Peter Lenz, and Hans-Ulrich Mösch. The transcription factors tec1 and ste12 interact with coregulators msa1 and msa2 to activate adhesion and multicellular development. *Molecular and cellular biology*, 34(12):2283–2293, 2014.

Zoé Van Havre, Nicole White, Judith Rousseau, and Kerrie Mengersen. Overfitting bayesian mixture models with an unknown number of components. *PloS one*, 10(7):e0131739, 2015.

Dootika Vats and Christina Knudson. Revisiting the gelman-rubin diagnostic. *arXiv preprint arXiv:1812.09384*, 2018.

Guangchuang Yu, Li-Gen Wang, Yanyan Han, and Qing-Yu He. clusterprofiler: an r package for comparing biological themes among gene clusters. *OMICS: A Journal of Integrative Biology*, 16(5):284–287, 2012. doi: 10.1089/omi.2011.0118.

Yasin Șenbabaoğlu, George Michailidis, and Jun Z Li. A reassessment of consensus clustering for class discovery. *bioRxiv*, page 002642, 2014a.

Yasin Șenbabaoğlu, George Michailidis, and Jun Z Li. Critical limitations of consensus clustering in class discovery. *Scientific reports*, 4(1):1–13, 2014b.
